# Exosomal Non‐Coding RNAs in Gastrointestinal Cancer Drug Resistance: A Systematic Review of Emerging Mechanisms and Clinical Implications

**DOI:** 10.1111/jcmm.71137

**Published:** 2026-05-08

**Authors:** Mohsen Sharif‐zak, Zahra Sadeghloo, Fateme Binayi, Stefania Nobili, Sara Ashtari, Amir Sadeghi, Nayeralsadat Fatemi

**Affiliations:** ^1^ Gastroenterology and Liver Diseases Research Center, Research Institute for Gastroenterology and Liver Diseases, Shahid Beheshti University of Medical Sciences Tehran Iran; ^2^ Basic and Molecular Epidemiology of Gastrointestinal Disorders Research Center, Research Institute for Gastroenterology and Liver Diseases Shahid Beheshti University of Medical Sciences Tehran Iran; ^3^ Department of Neuroscience, Psychology, Drug Research and Child Health – NEUROFARBA – Pharmacology and Toxicology Section University of Florence Firenze Italy

**Keywords:** drug resistance, exosomes, gastrointestinal neoplasms, neoplasm, RNA, untranslated

## Abstract

Gastrointestinal (GI) tumours are one of the most prevalent cancers globally. Even with normal GI function, individuals may develop neoplasms, highlighting the need to better understand the underlying causes of tumorigenesis. The emergence of tumour drug resistance represents the main reason for the failure of drug treatment; thus, it is imperative to investigate all the potential mechanisms of this very complex phenomenon. It is significant that exosomes and noncoding RNAs, such as microRNAs (miRNAs), long non‐coding RNAs (lncRNAs) and circular RNAs (circRNAs), originating from tumour cells, are linked to both GI drug resistance and the carcinogenesis and development of GI disease. Thus, we propose a systematic review of the literature to provide an overview of the current landscape concerning exosomal ncRNAs as diagnostic and prognostic biomarker resources in resistant GI cancer. We performed the current systematic review according to PRISMA guidelines and comprehensively explored PubMed, Web of Science, Scopus and Google Scholar databases to achieve the article search. Seventy‐six studies were included in the investigation, comprising 50 cohort studies and 26 nested case–control studies (between 1994 and 2025). Among all systematically reviewed exosomal ncRNAs, we primarily found molecules with prognostic significance and predictive relevance, and those that serve both purposes have been identified. Furthermore, among all analysed ncRNAs isolated from exosomes, miRNAs, lncRNAs and circRNAs have emerged as the most extensively studied and are proposed as potential tools for predicting resistance or sensitivity to GI cancer treatments. Our analysis offers a comprehensive overview of the current landscape concerning exosomal ncRNAs as potential clinical biomarkers for resistant GI tumours. However, despite extensive research efforts, the application of exosomal biomarkers in GI cancers is still in its infancy. None of the identified exosomal biomarkers have advanced beyond preclinical studies, and their applicability in clinical settings remains limited, largely due to challenges in clinical validation, standardisation and biomarker specificity.

## 1 Introduction

The efficacy of chemotherapy, the cornerstone of treatment for metastatic gastrointestinal (GI) cancers, is profoundly undermined by the pervasive challenge of drug resistance, which represents the principal driver of treatment failure and poor patient outcomes. Chemotherapy is the primary treatment for GI tumours that have metastasised [1, 2]. Surgery is in fact feasible only in oligometastatic GI cancers [3]. Traditional chemotherapy agents include 5‐fluorouracil (5‐FU), epirubicin, paclitaxel (PTX), irinotecan, oxaliplatin and cisplatin (CDDP) and others. However, the effectiveness of chemotherapy is limited, and the five‐year overall survival (OS) rate for metastatic GI cancer patients remains relatively low, ranging from 3% for pancreatic and hepatocellular carcinoma to 13% for colorectal cancer (www.cancer.org) [4, 5].

Even OS rates for patients with earlier stages of GI cancer remain unsatisfactory (www.cancer.org) [4, 5] despite the advancements in anticancer drug treatments., In addition, diagnostic efficiency is still below desired levels, particularly for patients with advanced tumours [6, 7].

With advancements in the early diagnosis and treatment of GI cancers, we recognise that an ideal biomarker could play a crucial role [8, 9]. Therefore, a primary objective is to identify novel and effective biomarkers for early diagnosis, prognosis prediction and optimal therapeutic targets for patients with GI cancer.

Exosomes are a class of extracellular vehicles (EVs) that range from 40 to 150 nm in diameter and possess specific surface molecular markers, including CD9, CD63, Hsp70 and TSP101 [10, 11].

Exosomes were first documented in sheep reticulocytes in 1983 [12]. Subsequent investigations have revealed that exosomes originate from a wide variety of cell types and can be detected in cell‐conditioned media as well as in various biological fluids, including serum, plasma, urine, saliva, ascitic fluid, cerebrospinal fluid and amniotic fluid [13, 14].

Exosomes in tumour cells have been shown to activate specific tumour resistance responses and promote the occurrence and progression of tumours [15]. Furthermore, numerous studies have reported that exosomes play a role in angiogenesis, metastasis, drug resistance, immune responses, cytokine secretion, apoptosis, cell proliferation and oncogenic transformation [16, 17, 18]. Exosomes perform these functions by interacting with the surface receptors of recipient cells, thereby transmitting a diverse cargo of biomolecules such as lipids, proteins, messenger RNAs (mRNAs) and non‐coding RNAs (ncRNAs) to those cells. Among this cargo, ncRNAs have emerged as particularly potent regulators of cellular phenotype. Their intercellular transfer provides a powerful mechanism for orchestrating complex biological responses, including the acquisition of therapeutic resistance [19]. Notably, ncRNAs are the components of exosomes that have garnered particular attention [20]. NcRNAs refer to molecules that lack protein‐coding regions and include microRNAs (miRNAs), long non‐coding RNAs (lncRNAs) and circular RNAs (circRNAs) [21, 22, 23].

Recent studies reveal that ncRNAs are essential regulators of numerous biological functions. They play a critical role in managing various physiological and pathological processes. Many diseases, particularly cancer, are closely associated with the deregulation of ncRNAs [24, 25, 26, 27]. Particularly, the dysregulation of ncRNAs plays a significant role in the development of drug resistance in cancer through multiple pathways including autophagy activation, improved epithelial cells transforming into mesenchymal cells and suppression of apoptosis [28, 29]. Moreover, ncRNAs hold significant potential as biomarkers and therapeutic targets for GI cancers due to their diverse expression profiles [30, 31, 32].

The connection between exosomes and ncRNAs is particularly fascinating. In 2007, Valadi et al. reported the first instance of RNAs being transported among cells via exosomes [33]. Their work catalysed the investigation of exosomal ncRNAs as crucial molecules in cell communication. This study indicates a correlation between resistance to oxaliplatin and the exosomal lncRNA CCAL in GI cancer. CCAL specifically interacts with the mRNA‐stabilising protein HuR (human antigen R), thereby enhancing the levels of β‐catenin and reducing apoptosis [34, 35]. Additionally, exosomal H19 contributes to chemotherapy resistance by activating the β‐catenin pathway, which competes with miR‐141 for endogenous RNA [36].

Despite the limited number of existing publications on this topic, a systematic review of the current literature is essential for a better understanding of the role of exosomal ncRNAs in drug resistance in GI cancers. This systematic review addresses a critical gap in the literature by providing the first comprehensive synthesis of the available evidence on all major exosomal ncRNAs (miRNAs, lncRNAs and circRNAs) involved in drug resistance across the entire spectrum of GI malignancies. Unlike previous reviews limited to specific ncRNA types or individual cancers, our objective is to systematically analyse the associations between ncRNA expression and therapeutic outcomes. Through this rigorous approach, this study aims to offer a holistic view of the field, evaluate the potential of these molecules as biomarkers for diagnosis and prognosis and identify convergent mechanistic pathways for future therapeutic targeting.

## 2 Materials and Methods

### 2.1 Design and Registration

We conducted this systematic review following the principles outlined in the Preferred Reporting Items for Systematic Reviews and Meta‐Analyses (PRISMA‐P) 2020 statement. The study protocol was rigorously registered with PROSPERO, the International Prospective Register of Systematic Reviews, under the unique code CRD420251055821.

### 2.2 Search Strategy

We conducted a comprehensive literature search across multiple databases, including PubMed, Web of Science, Scopus and Google Scholar, with the search concluding on October 18, 2025. Search strategies used official Medical Subject Headings (MeSH) without quotation marks to ensure compatibility with PubMed's indexing system and avoid spurious warnings, while maintaining full sensitivity. Final script for searching in the PubMed as follows; ((‘gastrointestinal neoplasms’[MeSH Terms] OR ‘colorectal neoplasms’[MeSH Terms] OR ‘pancreatic neoplasms’[MeSH Terms] OR ‘stomach neoplasms’[MeSH Terms] OR ‘liver neoplasms’[MeSH Terms] OR ‘oesophageal neoplasms’[MeSH Terms] OR ‘gastrointestinal cancer*’[Title/Abstract] OR ‘colorectal cancer*’[Title/Abstract] OR ‘pancreatic cancer*’[Title/Abstract] OR ‘gastric cancer*’[Title/Abstract] OR ‘stomach cancer*’[Title/Abstract] OR ‘liver cancer*’[Title/Abstract] OR ‘carcinoma, hepatocellular’[MeSH Terms] OR ‘oesophageal cancer*’[Title/Abstract]) AND (‘exosomes’[MeSH Terms] OR ‘exosomal’[Title/Abstract] OR ‘exosome‐derived’[Title/Abstract] OR ‘exosome’[Title/Abstract]) AND (‘rna, untranslated’[MeSH Terms] OR ‘rna, long noncoding’[MeSH Terms] OR ‘rna, circular’[MeSH Terms] OR ‘ncrnas’[Title/Abstract] OR ‘mirnas’[Title/Abstract] OR ‘lncrnas’[Title/Abstract] OR ‘circrnas’[Title/Abstract] OR ‘pirnas’[Title/Abstract]) AND (‘drug resistance, neoplasm’[MeSH Terms] OR ‘drug resistance’[Title/Abstract] OR ‘chemoresistance’[Title/Abstract] OR ‘therapy resistance’[Title/Abstract]) AND 1900/01/01:2025/10/18[Date—Publication]). A copy of an example search strategy for the Scopus and Web of Science are provided in the File S1. Furthermore, Grey literature (i.e., unpublished literature) was searched using the national grey literature collection (https://allcatsrgrey.org.uk). Preprinted servers medRxiv (https://www.medrxiv.org) and PsyArXiv (https://psyarxiv.com) were also searched. An incognito Google Scholar search was undertaken with the first 200 results of returns reviewed for inclusion. These were searched using the EThOS (https://ethos.bl.uk). Additionally, reference lists of potentially eligible papers were manually reviewed to ensure no relevant studies were missed. Only studies published in English language were included to maintain relevance and applicability without date limitations. The comprehensive search strings and the specific scripts used for each database are detailed in the File S1.

### 2.3 Inclusion and Exclusion Criteria

The eligibility criteria for study selection were established based on the PECOs framework [37] and aligned with the PRISMA guidelines. Therefore, in accordance with the PECOs framework and PRISMA guidelines, studies were included if they met the following criteria: (P) Population: Human subjects diagnosed with GI cancers, including but not limited to colorectal, gastric, pancreatic, oesophageal and hepatocellular carcinomas. (E) Exposure: Studies that evaluated the expression of exosomal ncRNAs, specifically miRNAs, lncRNAs, or circRNAs, in relation to therapeutic resistance. (C) Comparator: Studies that compared patients with drug‐resistant GI cancers to those who were drug‐sensitive or responders to treatment, enabling a comparative analysis of exosomal ncRNA expression patterns. (O) Outcomes: Studies that assessed the role of exosomal ncRNAs as diagnostic or prognostic biomarkers for identifying or predicting resistance to therapy in GI cancers. (S) Study Design: Original research studies including cohort studies, case–control studies and cross‐sectional studies were considered eligible. Studies published in English with full‐text availability were included, with no restriction on publication date. Preprints and grey literature were also eligible if they provided sufficient methodological and outcome details.

We excluded studies that involved animal models, cell lines, or purely in vitro experiments without validation in human samples. Additionally, studies that did not specifically address therapeutic resistance in GI cancers or did not focus on exosomal forms of ncRNAs were excluded. Reviews, editorials, commentaries, conference abstracts without accessible full texts, letters to the editor and case reports were also excluded. Furthermore, studies investigating ncRNAs outside the context of drug resistance, or those evaluating circulating but non‐exosomal ncRNAs, were not considered eligible. Only English‐language publications were included to ensure clarity and applicability.

### 2.4 Study Selection

The search results were managed and screened using EndNote software (version 20.2.1). After removing duplicate records, two independent reviewers (MS and FB) screened the titles and abstracts to assess their relevance. Full‐text articles of potentially eligible studies were then thoroughly reviewed to confirm inclusion. Any disagreements between the reviewers were resolved through discussion, and when necessary, a third reviewer (HS) was consulted to reach a consensus. This process resulted in a high level of inter‐rater reliability, as demonstrated by a Kendall's coefficient of 0.92 (*p* < 0.01). The overall study selection process was visually summarised in a PRISMA 2020 flow diagram.

### 2.5 Quality Assessment

The methodological quality of all included studies was independently assessed by two reviewers (MS and HS). Any discrepancies in judgement were resolved through discussion and, when necessary, in consultation with a third reviewer (FB) to reach consensus. The quality of each study was assessed using the Critical Appraisal Skills Programme (CASP) checklist by three reviewers (Tables S1 and S2). As part of the screening process and quality assessment, conflicts were resolved with the fourth reviewer (NF) through discussions or consultation.

### 2.6 Data Extraction

Following the selection of eligible studies, two independent investigators extracted relevant data from the included studies into a pre‐designed Excel spreadsheet. For each study, the following characteristics were recorded: year of publication, authors, exosome isolation, detection method, clinical patients, exosomal ncRNAs, exosomes diameter range (nm), clinical sample, clinical patients, drug, expression pattern of exosomal ncRNAs, role in chemoresistance, targets or pathways, biomarker type (prognostic or diagnostic) and mechanism of regulation. The summarised data are presented in Tables 1, 2, 3, 4, 5.

**TABLE 1:** Exosomal ncRNAs (miRNAs, lncRNAs and circRNAs) involved in colorectal cancer drug resistance.

NcRNA	Diameter size (nm)	Exosome isolation	Detection methods	Clinical patients	Sample	Drug	Expression pattern	Role in chemoresistance	Targets or pathways	Biomarker type	Mechanisms	References
miR‐24‐3p	50–100	Ultrafiltration	Nanosight LM20 system/Western Blotting	CRC	Tissue	Methotrexate	Up	Inducing	CDX2/HEPH axis	Predictive	Downregulating the CDX2/HEPH axis	Zhang et al. [38]
miR‐208b	100	Gradient centrifugation	qPCR/Western Blotting	CRC	Serum	Oxaliplatin	Up	Inducing	PDCD4	Prognostic	Upregulating PDCD4	Ning et al. [39]
miR‐92a‐3p	50–100	Ultracentrifugation/Precipitation	Nanosight analysis/Western Blotting	CRC	Serum, Tissue	5‐Fluorouracil, oxaliplatin	Up	Inducing	FBXW7 and MOAP1/Wnt/β‐catenin pathway	Predictive	Activating the Wnt/β‐catenin pathway/Inhibiting mitochondrial apoptosis by directly inhibiting FBXW7 and MOAP1	Hu et al. (2019)
miR‐196b‐5p	Not available	Precipitation	qPCR	CRC	Serum, Tissue	5‐Fluorouracil	Up	Inducing	STAT3 signalling pathway	Prognostic	Activating the STAT3 signalling pathway	Ren et al. [40]
miR‐200b‐3p	40–150	Ultracentrifugation	Nanoparticle tracking analysis/Western Blotting/RNA‐seq	CRC	Tissue	5‐Fluorouracil	Down	Reversing	ZEB1 and E2F3	Predictive	Upregulating ZEB1 and E2F3	Gong et al. [41]
miR‐625‐3p	Not available	Ultracentrifugation	Western Blotting	CRC with tumour invasion margins more significant than 10 cm	Tissue	Oxaliplatin, fluorouracil, docetaxel, irinotecan, mitomycin	Up	Reversing	UGBP Elav‐like family member 2/WW Domain Containing Oxidoreductase	Predictive	Inhibiting the UGBP Elav‐like family member 2/WW Domain Containing Oxidoreductase pathway	Zhang et al. [42]
miR‐181d‐5p	42–197	Centrifugation	Flow Nano Analyser/Western Blotting	CRC	Tissue	5‐Fluorouracil	Up	Inducing	Neurocalcin δ (NCALD)	Prognostic	Targeting neurocalcin δ (NCALD) by miR‐181d‐5p inhibits the sensitivity of CRC cells to 5‐Fluorouracil	Pan et al. [43]
miR‐590‐3p	50–100	Centrifugation/Precipitation	Nanosight analysis/Western Blotting	Chemo resistant and chemo sensitive patients	Serum, Tissue	Radiotherapy	Up	Inducing	CLCA4/PI3K/Akt signalling pathway	Predictive	Upregulating the CLCA4‐dependent PI3K/Akt signalling pathway	Chen et al. [44]
miR‐184, miR‐100, miR‐92a, miR‐16,	100	Centrifugation/Precipitation	Nanoparticle tracking analysis/Western Blotting	Chemo resistant and chemo sensitive patients	Serum	Oxaliplatin	Up	Inducing	PI3K‐AKT signalling pathway, AMPK signalling pathway, and FoxO signalling pathway	Predictive	Activating the PI3K‐AKT, AMPK and FoxO signalling pathways	Han et al. [45]
miR‐30e, miR‐144‐5p and let‐7i	100	Centrifugation/Precipitation	Nanoparticle tracking analysis/Western Blotting	Chemo resistant and chemo sensitive patients	Serum	Oxaliplatin	Down	Reversing	PI3K‐AKT signalling pathway, AMPK signalling pathway and FoxO signalling pathway	Predictive	Activating the PI3K‐AKT, AMPK and FoxO signalling pathways	Han et al. [45]
miR‐125b	50–100	Ultracentrifugation/TEM	Flow cytometry	Chemo‐resistant patients in advanced and recurrent CRC	Serum	mFOLFOX6	Up	Reversing	Not available	Prognostic	Not available	Yagi et al. [46]
miR‐21–5p, miR‐1246, miR‐1229‐5p, miR‐96‐5p	50–150	Ultracentrifugation/TEM	qPCR/Western Blotting	CRC	Serum	5‐fluorouracil, oxaliplatin	Up	Inducing	PI3K‐Akt signalling pathway, FoxO signalling pathway and autophagy pathway	Predictive	Activating the PI3K‐Akt, FoxO and autophagy pathway	Jin et al. [47]
LncRNA FAL1	Not available	Differential centrifugation	Bicinchoninic Acid kit	Non‐recurrence and recurrence	Tissue	Oxaliplatin	Up	Inducing	TRIM3‐dependent Beclin1	Prognostic	Promoting TRIM3‐dependent Beclin1 polyubiquitination/Suppressing oxaliplatin‐induced autophagic cell death	Zhu et al. [48]
LncRNA CACClnc	100–200	Centrifugation	Nano sight analysis/Western Blotting	Non‐recurrence and recurrence	Plasma	Oxaliplatin, 5‐Fluorouracil	Up	Inducing	Y‐box binding protein 1 and U2AF65 (a subunit of U2AF splicing factor)	Prognostic	Promoting the interaction between Y‐box binding protein 1 and U2AF65	Zhang et al. [49]
LncRNA PGM5‐AS1	50–150	Ultracentrifugation/Precipitation	qPCR	Non‐recurrence and recurrence	Tissue	Oxaliplatin	Down	Inducing	NME1/PAEP/SRSF3	Prognostic	Upregulating NME1 expression/Activating alternate splicing to downregulate PAEP expression by recruiting SRSF3	Hui et al. [50]
LncRNA CCAL	100	Ultrafiltration	Nanoparticle tracking analysis/qPCR	CRC	Tissue	Oxaliplatin	Up	Inducing	β‐catenin signalling pathway	Predictive	Activating the β‐catenin signalling pathway	Deng et al. [51]
LncRNA H19	30–150	Gradient centrifugation	Nanoparticle tracking/RNA‐seq/Western Blotting	CRC	Tissue	Oxaliplatin	Up	Inducing	β‐catenin signalling pathway	Predictive	Activating the β‐catenin signalling pathway	Ren et al. [52]
LINC00355	50–185	Ultracentrifugation/Precipitation	Nanoparticle tracking analysis/Western Blotting	CRC	Tissue	5‐fluorouracil, oxaliplatin, cisplatin	Up	Inducing	CRKL and miR‐34b‐5p	Predictive	Upregulating CRKL expression via inhibiting the expression of miR‐34b‐5p	Hu et al. [53]
LncRNA HOTTIP	NA	Precipitation	Nano sight analysis/Western Blotting	Chemoradiotherapy responders and non‐responders	Serum, Tissue	Mitomycin	Up	Inducing	KPNA3	Prognostic	Upregulating KPNA3	Chen et al. [44]
LncRNA UCA1	30–150	Ultracentrifugation	q‐PCR/Western Blotting	Responders and non‐responders	Serum	Cetuximab	Up	Inducing	Not available	Predictive	UCA1, leading to dysregulation of the signalling pathway in cetuximab resistance	Yang et al. [54]
Circ0067557	100	Ultracentrifugation	Dynamic light scattering/Western Blotting	CRC	Tissue	5‐Fluorouracil, Oxaliplatin	Up	Inducing	Lin28A and Lin28B	Prognostic	Upregulating Lin28A and Lin28B	Yang et al. [55]
Circ0001610	30–150	Centrifugation	Nanoparticle tracking analysis/Western Blotting	CRC	Serum	Oxaliplatin	Up	Inducing	OXPHOS	Prognostic	Upregulating PGC‐1a‐dependent OXPHOS activity	Deng et al. [56]
Circ0006174	100	Centrifugation/Precipitation	qPCR	CRC	Tissue	Doxorubicin	Up	Inducing	CCND2 and miR‐1205	Predictive	Upregulating CCND2 expression through sponging miR‐1205	Zhang et al. [42]
Circ0000338	150	Centrifugation/Precipitation	Nanoparticle tracking analysis/qPCR/Western Blotting	Responders and non‐responders	Tissue	5‐Fluorouracil	Up	Inducing	miR‐217 and miR‐485‐3p	Predictive	Downregulating miR‐217 and miR‐485‐3p	Zhao et al. [57]
CircFBXW7	100 ± 60	Centrifugation/Precipitation	qRT‐PCR/Western Blotting	Resistant and ‐sensitive groups	Tissue	Oxaliplatin	Down	Reversing	miR‐18b‐5p	Predictive	circ‐FBXW7 sponges miR‐18b‐5p, leading to reduced drug resistance	Xu et al. [58]
Circ0005963	50–150	Ultracentrifugation/TEM	Nanoparticle tracking analysis/Western Blotting	Resistant and ‐sensitive groups	Serum	Oxaliplatin	Up	Inducing	PKM2//miR‐122	Predictive	Upregulating PKM2	Wang et al. [59]
Circ0000338	60–100	Ultracentrifugation/TEM	Zetasizer Nano ZS system/Western Blotting	Resistant and Sensitive groups	Serum	FOLFOX	Up	Inducing	Not available	Predictive	Not available	Hon et al. [60]
Circ0004771	100	Differential centrifugation	Nanoparticle tracking analysis/Western Blotting/Flow cytometry	Resistant and ‐sensitive groups	Serum, Tissue	5‐Fluorouracil	Up	Inducing	miR‐653/ZEB2 signalling pathway	Predictive, Prognostic	Modulating miR‐653/ZEB2 signalling pathway	Qiao et al. [61]
CircATG4B	100–300	Centrifugation	Nanoparticle tracking analysis/qPCR/Western Blotting	CRC	Tissue	Oxaliplatin	Up	Inducing	TMED10 and ATG4B	Prognostic	Preventing TMED10 from binding to ATG4B/Promoting autophagy	Pan et al. [62]
CricN4BP2L2	110	Ultracentrifugation/Precipitation	qNano/qPCR	CRC	Tissue	Oxaliplatin	Up	Inducing	EIF4A3/PI3K/AKT/mTOR axis	Predictive	Activating the PI3K/AKT/mTOR axis by binding to EIF4A3	Qu et al. [63]
Circ0094343	75–150	Ultrafiltration	Nanoparticle tracking analysis/Western Blotting	Metastasis and non‐metastasis group/Resistant and ‐sensitive groups	Tissue	5‐fluorouraci, oxaliplatin, doxorubicin	Down	Reversing	miR‐766‐5p	Predictive	Inhibiting CRC proliferation/glycolysis/chemoresistance via miR‐766‐5p/TRIM67	Li and Li [64]

### 2.7 Synthesis of Results

A qualitative synthesis of the included studies was conducted due to significant methodological and clinical heterogeneity across the evidence base. The reviewed studies did not provide standardised effect sizes, comparable treatment groups, or uniform outcome measures that would permit statistical pooling or meta‐analysis. As a result, a quantitative synthesis was not feasible, and this review is limited to a descriptive analysis of the findings. The lack of consistent reporting on key variables – such as exosomal ncRNA expression patterns, resistance definitions and outcome measures – precluded the use of meta‐analytical techniques. Therefore, this work remains a systematic review without meta‐analysis, in accordance with PRISMA guidelines.

## 3 Results

### 3.1 Search Result, Study Characteristics and Quality Assessment

**FIGURE 1:**
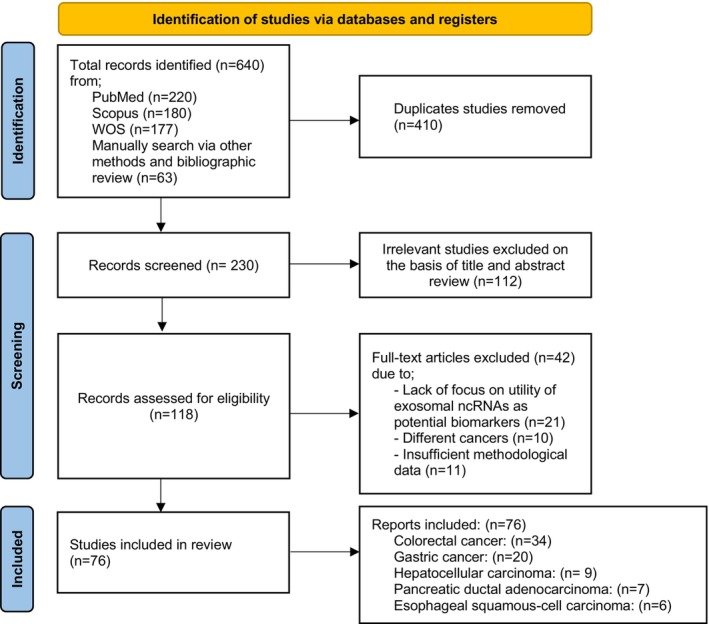
Literature search results and the screening process based on PRISMA 2020 flowchart.

The systematic literature search identified a total of 640 records across multiple sources: PubMed (*n* = 220), Scopus (*n* = 180), Web of Science (*n* = 177) and manual searches or bibliographic reviews (*n* = 63). Following deduplication, 410 duplicate records were removed. An additional 112 irrelevant studies were excluded during title and abstract screening, resulting in 118 records being assessed for eligibility. Of these, 42 full‐text articles were excluded for the following reasons: lack of focus on the utility of exosomal ncRNAs as potential biomarkers (*n* = 21), investigation of non‐gastrointestinal cancers (*n* = 10), or insufficient methodological data (*n* = 11). Ultimately, 76 studies met the inclusion criteria and were included in the qualitative synthesis. These studies examined the role of exosomal ncRNAs in drug resistance across various gastrointestinal cancers, including colorectal cancer (*n* = 34), gastric cancer (*n* = 20), hepatocellular carcinoma (*n* = 9), pancreatic ductal adenocarcinoma (*n* = 7) and oesophageal squamous‐cell carcinoma (*n* = 6). A complete overview of the literature search and study selection process is illustrated in Figure 1, based on the PRISMA 2020 flowchart. Additionally, baseline characteristics of the included manuscripts are presented in Tables 1, 2, 3, 4, 5. There were 76 studies that analysed the association between exosomal ncRNAs and drug resistance in GI cancers, 50 cohort studies 26 nested case–control studies were included, these studies were published between 1994 and 2024. A quality appraisal determined that all inquiries were high‐quality studies.

### 3.2 Exosome‐Derived ncRNAs Influence Drug Resistance in GI Cancers

The pivotal role of exosome‐derived ncRNAs in drug resistance of colorectal cancer (CRC), pancreatic ductal adenocarcinoma (PDAC), hepatocellular carcinoma (HCC), oesophageal squamous‐cell carcinoma (ESCC) and gastric cancer (GC) is comprehensively explored in this review (Tables 1, 2, 3, 4, 5). Notably, the role of exosomal lncRNAs in HCC remains severely understudied. Our exhaustive literature analysis yielded only one study addressing this topic, which signifies a significant gap in the current research landscape.

#### 3.2.1 Drug Resistance Mediated by Exosomal miRNAs in GI Cancers

##### 3.2.1.1 Colorectal Cancer

Recent advances have underscored the pivotal role of exosomal ncRNAs, particularly miRNAs, in mediating drug resistance in CRC. Here, we identified 15 studies that focus on miRNAs (Table 1). Analysis of these studies reveals two dominant themes: the convergence of multiple miRNAs on conserved intracellular signalling pathways and the critical role of the tumour microenvironment (TME) as a source of these resistance‐mediating molecules.

In a Chinese cohort of 116 participants, Ning et al. reported a significant upregulation of miR‐208b (*p* < 0.001) in the serum of oxaliplatin‐resistant CRC patients. This miRNA suppresses programmed cell death 4 (PDCD4), promoting the expansion of regulatory T cells (Tregs) and enhancing immunosuppressive responses [39]. Similarly, Yagi et al. showed that miR‐125b is moderately upregulated (*p* < 0.01) in the serum of 134 Japanese patients resistant to the mFOLFOX6 (oxaliplatin plus leucovorin plus 5‐fluorouracil) regimen [46]. This miRNA is also associated with *KRAS* mutation status. Furthermore, an upregulated expression (*p* < 0.01) of combinatorial miRNA panels, including miR‐21–5p, miR‐1246, miR‐1229‐5p and miR‐96‐5p, has been identified in the serum of 43 Chinese patients with resistance to 5‐FU and oxaliplatin [47]. In another study of 186 Chinese patients with oxaliplatin resistance, miR‐184 (*p* < 0.05), miR‐100 (*p* < 0.0001), miR‐92a (*p* < 0.0001) and miR‐16 (*p* < 0.05) were upregulated, while miR‐30e (*p* < 0.0001), miR‐144‐5p (*p* < 0.0001) and let‐7i (*p* < 0.0001) were downregulated. These miRNAs collectively target multiple signalling pathways, including PI3K/AKT, FoxO, autophagy and AMPK [45]. The modulation of the PI3K/AKT pathway appears to be a convergent mechanism in CRC resistance, as it was implicated in multiple independent studies.

Beyond the actions of tumour‐derived exosomes, a significant number of the identified studies reveals the pivotal role of the TME. Specifically, cancer‐associated fibroblasts (CAFs) were repeatedly identified as a primary source of exosomal miRNAs that actively confer chemoresistance. Among CAF‐derived miRNAs, Zhang et al. reported a significant upregulation (*p* < 0.05) of miR‐24‐3p, which confers methotrexate resistance by targeting the CDX2/HEPH axis [38]. Additionally, miR‐625‐3p, also derived from CAFs, disrupts the CELF2/WWOX tumour suppressor axis, leading to broad resistance against multiple chemotherapeutics, including oxaliplatin, 5‐FU, docetaxel, irinotecan and mitomycin [42]. Likewise, Hu et al., showed that miR‐92a‐3p is highly upregulated in both serum (*p* < 0.01) and tissues (*p* < 0.001), promoting resistance to 5‐FU/L‐OHP via Wnt/β‐catenin activation and inhibiting apoptosis‐related targets such as FBXW7 and MOAP1 [96]. miR‐196b‐5p, which is upregulated in the serum (*p* < 0.01) and tissues (*p* < 0.05) of 110 Chinese patients, promotes resistance through the activation of STAT3 [40]. Meanwhile, miR‐181d‐5p, studied in 30 Chinese patients, exerts its resistance‐inducing effect by mediating m6A upregulation (*p* < 0.001) and inhibiting the expression of NCALD [43]. Chen et al., demonstrated that miR‐590‐3p is upregulated in both serum (*p* < 0.01) and tissues (*p* < 0.05) of CRC patients, enhancing radioresistance by targeting CLCA4, leading to the activation of the PI3K/AKT signalling pathway [44]. In a multinational cohort of 120 patients, Xu et al. reported the differential expression of exosomal miRNAs in chemotherapy‐naive RAS/BRAF WT mCRC patients treated with cetuximab or panitumumab. Specifically, miR‐487‐5p, miR‐4454, miR‐664a‐3p, miR‐500a‐5p, miR‐500b‐5p, miR‐218‐5p, miR‐143‐5p and miR‐654‐3p were upregulated (*p* < 0.05) in good responders, reversing resistance [97]. Conversely, miR‐338‐3p, miR‐7976, miR‐452‐5p, miR‐3200‐3p and miR‐330‐3p were downregulated (*p* < 0.05) in good responders, inducing resistance. These miRNAs target STXBP1, EPS8, FOS, GRB10, PAK1, MAPK4, STAT1, MAP2K1, PDGFA, FGFR2 and AKT3, modulating post‐transcriptional resistance mechanisms that regulate the TP53 cascade and escape from apoptosis [97]. Similarly, Gong et al. revealed that the loss of miR‐200b‐3p (*p* < 0.01) in exosomes derived from hypoxic CAFs, as observed in a small Chinese study involving five patients, results in increased expression of ZEB1 and E2F3, thereby reducing sensitivity to 5‐FU [41]. Yan et al. showed in a Chinese cohort of chemo‐resistant and chemo‐sensitive CRC patients that miR‐224‐5p is significantly downregulated (*p* < 0.05) in serum exosomes, conferring 5‐FU resistance by targeting S100A4 and modulating calcium‐related pathways to enhance malignant properties [98]. Additionally, in a study of 50 CRC patients in China, Zhao et al. reported a significant upregulation of miR‐223‐3p (*p* ≤ 0.001) in serum and tissue samples. By targeting NF2 and disrupting the Hippo signalling pathway, this miRNA facilitates the malignant properties of CRC and induces resistance to 5‐FU [99]. Therefore, these findings in CRC illustrate a complex landscape where exosomal miRNAs, originating from both cancer cells and the surrounding stroma, coordinate drug resistance by hijacking a core set of cell survival and signalling pathways.

##### 3.2.1.2 Pancreatic Ductal Adenocarcinoma

The role of miRNAs in the onset and advancement of PDAC has made them a highly relevant target for biomarker research. We found five studies specifically concentrating on miRNAs (Table 2). Ran et al. studied 65 Chinese patients (including 6 with primary pancreatic cancer, 13 treated with neoadjuvant gemcitabine (GEM) chemotherapy and 46 without neoadjuvant chemotherapy) and found that upregulation of miR‐3173‐5p (*p* < 0.001) in tumour tissues after chemotherapy enhanced resistance to GEM [65]. Significantly, CAF exosome‐derived miR‐3173–5p overexpression sponged ACSL4, suppressed ferroptosis and reduced Fe^2+^ and lipid ROS concentrations after GEM exposure, suggesting its possible involvement in cancer progression [65]. In another study conducted by Xu and colleagues involving Chinese pancreatic cancer patients (*n* = 8), significant downregulation of miR‐124 (*p* < 0.001) was observed, alongside elevated expression of EZH2 (*p* < 0.001) in pancreatic adenocarcinoma tissues. Their findings further demonstrated that exosome derived from bone marrow mesenchymal stem cells (BM‐MSCs) can effectively transport miR‐124 to pancreatic cancer cells. These miR‐124‐enriched exosomes inhibited tumour cell proliferation, metastasis and epithelial‐mesenchymal transition (EMT), while simultaneously increasing sensitivity to 5‐FU chemotherapy [58]. Among the five key radiobiological principles known as the 5Rs, tumour repopulation is particularly significant, as it often results in unsuccessful treatment outcomes. In a study involving 12 Chinese patients, Jiang and colleagues observed elevated expression of exosomal miR‐194‐5p (*p* < 0.001) derived from irradiated, dying tumour cells. Their findings demonstrated that miR‐194‐5p significantly inhibits the proliferation of pancreatic cancer (PC) cells. Furthermore, the forced expression of this miRNA suppressed cellular migration and invasion while paradoxically enhancing the tumour repopulation capacity [66]. They also found PGE2, a lipid metabolite recognised as a key mediator of tumour repopulation, following chemoradiation that accelerated tumour cell proliferation. However, the addition of PGE2 to the tumour cells immediately post radiation inhibited the viability of those cells. In this study, they also determined that aspirin reduced PGE2 levels in the supernatant of the irradiated PC cells and decreased the number of exosomes released from these cells. Moreover, aspirin was found to influence exosomes and miRNAs; specifically, miR‐194‐5p was significantly downregulated (*p* < 0.001) in exosomes derived from aspirin‐treated irradiated cells [66]. An experimental study involving 10 tissues from Chinese PC patients demonstrated increased expression of miR‐106b (*p* < 0.01) led to heightened resistance to GEM, with CAF‐secreted exosomes facilitating the delivery of miR‐106b to PC cells [67]. Since miR‐106b has a binding site for TP53INP1, a crucial tumour suppressor, it directly binds to this target, resulting in downregulation. This molecular interaction has been shown to promote GEM resistance in PC cells. Their findings revealed that elevated miR‐106b expression in PC cells strongly decreased TP53INP1 protein levels, subsequently increasing GEM resistance [67]. In another study conducted on tissue samples from 45 Japanese patients with PDAC, it was reported that high expression levels of miR‐155 (*p* < 0.01) promote exosome release and enhance miR‐155 levels within exosomes, ultimately contributing to GEM resistance [68]. miR‐155 exerts anti‐apoptotic effects by targeting TP53INP1, a pro‐apoptotic gene that is activated by p53 under stress conditions. Elevated miR‐155 expression increases both the protein and mRNA levels of TP53INP1 (*p* < 0.01), further contributing to GEM resistance in PDAC cells [68].

**TABLE 2:** Exosomal ncRNAs (miRNAs, lncRNAs and circRNAs) involved in pancreatic ductal adenocarcinoma drug resistance.

NcRNA	Diameter size (nm)	Exosome isolation	Detection methods	Clinical patients	Sample	Drug	Expression pattern	Role in chemoresistance	Targets or pathways	Biomarker type	Mechanisms	References
miR‐3173–5p	NA	Centrifugation and Precipitation	Western Blotting and Immunofluorescence and NTA	PDAC and health tumour after surgical	Tissue	Gemcitabine	UP	Inducing	miR‐3173–5p/ACSL4 axis	Prognostic	Suppressing ferroptosis through the miR‐3173–5p/ACSL4 axis and reducing Fe2+ and lipid ROS concentrations	Qi et al. [65]
miR‐124	30–130	Centrifugation and Precipitation	Western Blotting and Annexin V‐propidium iodide (PI) staining/NTA	PDAC and health tumour after surgical	Tissue	5‐Fluorouracil	Down	Reversing	miR‐124/EZH2 axis	Predictive	Suppressing the proliferation, metastasis and epithelial mesenchymal transition (EMT)	Xu et al. [58]
miR‐194‐5p	NA	Ultracentrifugation	Western Blotting/NAT	PDAC and health tumour after surgical	Tissue and blood	Radiation or radiation plus GW4869	Up	Inducing	miR‐194‐5p/PGE2 axis	Prognostic	Suppressing cellular migration and invasion and enhancing tumour repopulation capacity	Jiang et al. [66]
miR‐106b	80–130	Centrifugation	Western Blotting/qNano	PDAC without preoperative radiotherapy or chemotherapy.	Tissue	Gemcitabine	Up	Inducing	miR‐106b/TP53INP1 axis	Prognostic	Downregulation of TP53INP1 via binding with miR‐106b	Fang et al. [67]
miR‐155	89	Centrifugation	Western Blotting/NTA	PDAC after surgical with chemotherapy or post‐recurrence chemotherapy but not preoperative chemotherapy	Tissue and blood	Gemcitabine	Up	Inducing	miR‐155/TP53INP1 axis	Prognostic	Downregulation of TP53INP1 via binding with miR‐155	Mikamori et al. [68]
LncRNA UCA1	96.1 nm (PSC‐EXO) and 106.5 nm (HPSC‐EXO)	Centrifugation	Western Blotting/Flow cytometry/qNano	PDAC after surgical (10 cases of well differentiated, 14 cases of intermediate stage and 11 cases of poorly differentiated)	Tissue	Gemcitabine	Up	Inducing	LncRNA UCA1/EZH2 axis	Predictive	Recruiting EZH2 to the promoter of SOCS3 and downregulation SOCS3 expression	Chi et al. [69]
CircZNF91	NA	Ultracentrifugation	Western Blotting/qNano	PDAC undergoing surgical treatment	Tissue and blood	Gemcitabine	Up	Inducing	CircZNF91/miR‐23b‐3p/SIRT1 axis	Prognostic	Sponging miR‐23b‐ 3p to elevate SIRT1 expression, and deacetylation‐dependent stabilisation of HIF‐1α protein, and increasing the stability of HIF‐1α protein and subsequent activation of glycolytic pathways	Zeng et al. [70]

##### 3.2.1.3 Hepatocellular Carcinoma

Four studies evaluated the association between exosomal miRNAs expression and HCC resistance (Table 3). Bioinformatics analysis of a study conducted by Tang and colleagues, which involved six patients from China, revealed that blood exosome‐derived miR‐30d‐5p is highly expressed in HCC (*p* < 0.05) and promotes stemness and GEM resistance [71]. Additionally, the results of quantitative reverse transcription polymerase chain reaction (qRT‐PCR) indicated that SOCS3 expression was significantly lower in HCC cells (*p* < 0.05), suggesting a tumour‐suppressive role in the biological processes of HCC progression. On the basis of a dual luciferase assay demonstrating that miR‐30d‐5p can bind to SOCS3, the overexpression of miR‐30d‐5p was found to downregulate SOCS3 expression [71]. Weis et al. conducted a study on 18 tissue samples from Chinese patients, which demonstrated that the expression of miR‐135a‐5p was significantly increased (*p* < 0.01) and correlated with resistance to doxorubicin hydrochloride (Dox) [72]. Moreover, this study identified the protein expression of vesicle‐associated membrane protein 2 (VAMP2), another novel target, inhibited by miR‐135a‐5p (*p* < 0.05) that mediates Dox‐induced apoptosis in HCC [72]. On the other hand, it has been shown that the hepatitis B virus (HBV) induces the overexpression of the exosomal miR‐135a‐5p, thereby promoting survival and growth of HCC cells and preventing apoptosis through the miR‐135a‐5p/VAMP2 pathway [72]. Lou et al. revealed that the upregulation of miR‐199a‐enriched exosomes derived from adipose tissue mesenchymal stem cells (*p* < 0.01) enhances the sensitivity of HCC to doxorubicin in tissue samples from three Chinese patients [73]. Because of their ability to facilitate intercellular communication and transport exogenous molecules, adipose tissue‐derived mesenchymal stem cells (AMSC)‐derived exosomes modified with miR‐199a‐3p can efficiently transfer this miRNA between AMSCs and HCC cells. This process enhances the sensitivity of HCC cells to chemotherapeutic drugs by modulating the mTOR signalling pathway [73]. Lu and colleagues conducted a study in China in which subcutaneous adipose tissues were collected from three female patients (aged 32, 28 and 41) undergoing tumescent liposuction. The researchers subsequently isolated exosomes from the AMSCs and utilised them for miR‐122 delivery [74]. AMSCs transfected with miR‐122 efficiently incorporate miR‐122 into secreted exosomes, facilitating intercellular communication between AMSCs and HCC cells. Furthermore, exosomes derived from miR‐122 were upregulated (*p* < 0.05) and enhanced the sensitivity of HCC cells to sorafenib by promoting apoptosis and inducing cell cycle arrest [74]. Additionally, their findings revealed that miR‐122‐modified adipose tissue‐derived MSCs upregulate apoptosis‐related genes, such as Caspase 3 and Bax (Bcl‐2 Associated X Protein), while downregulating cyclin G1 (CCNG1), a disintegrin and metalloprotease 10 (ADAM10) and insulin‐like growth factor receptor 1 (IGF1R) in HCC cells (*p* < 0.05). This miRNA‐mediated alteration of miR‐122‐target gene expression enhances the susceptibility of cancer cells to chemotherapy [74].

**TABLE 3:** Exosomal ncRNAs (miRNAs, lncRNAs and circRNAs) involved in hepatocellular carcinoma drug resistance.

NcRNA	Diameter size (nm)	Exosome isolation	Detection methods	Clinical patients	Sample	Drug	Expression pattern	Role in chemoresistance	Targets or pathways	Biomarker type	Mechanisms	References
miR‐30d‐5p	100	Centrifugation	Western Blotting	HCC before surgery, without any preoperative treatments	Tissue, Serum	Gemcitabine	Up	Inducing	miRNA‐30d‐5p/SOCS3 axis	Predictive	Decreasing SOCS3 expression via binding to miRNA‐30d‐5p	Tang et al. [71]
miR‐135a	30 to 150	Ultracentrifugation	Western Blotting, dynamic light scattering (DLS)	HCC after surgical with Hepatitis B virus	Tissue	Doxorubicin	Up	Inducing	miR‐135a‐5p/VAMP2 pathway	Predictive	Inhibiting VAMP2 by miR‐135a‐5p	Wei et al. [72]
miR‐199a	80.0 ± 1.9	Precipitation	Western Blotting/NTA	HCC	Tissue	Doxorubicin	Up	Reversing	miR‐199a/mTOR pathway	Predictive	Suppressing of mTOR through miR‐199a	Lou et al. [73]
miR‐122	NA	Precipitation	Western Blotting	HCC	Tissue	Sorafenib	UP	Reversing	miR‐122 and targeting apoptosis‐related genes	Predictive	Upregulation Caspase 3 and Bax, downregulation CCNG1, ADAM10 and IGF1R by miR‐122	Lou et al. [74]
CircDCAF8	100	Centrifugation	Western Blotting/NTA	HCC patients during operations	Tissue	Regorafenib	Up	Inducing	circDCAF8/miR‐217/NAP1L1 axis	Prognostic	Suppresses miR‐217 level and increasing levels NAP1L1 via circDCAF8	Gong et al. [75]
CircUPF2	140.9 nm (Exo‐SR), 137.4 nm (Exo‐Norm)	Centrifugation	Western Blotting/NTA	HCC and healthy	Tissue, blood	Sorafenib	Up	Inducing	circZNF91/miR‐23b‐3p axis	Prognostic	Suppression of miR‐23b‐3p by binding to circZNF91	Dong et al. [76]
CircCCAR1	50 to 100	Precipitation	Western Blotting	HCC and healthy	Tissue	Anti‐PD1 immunotherapy	Up	Inducing	circCCAR1/miR‐127‐5p/WTAP axis	Prognostic	Sponging miR‐127‐5p and upregulation its target WTAP by interaction with CircCCAR1, and increasing circCCAR1 stability through a combination WTAP with IGF2BP3	Hu et al. [77]
CircG004213	30 to 150	Centrifugation	Western Blotting	HCC	Tissue, blood	Cisplatin	Up	Reversing	circRNA‐G004213/miR‐513b‐5p/PRPF39 axis	Prognostic	Upregulation miR‐513b‐5p and PRPF39 through circ‐G004213	Qin et al. [78]

##### 3.2.1.4 Gastric Cancer

We found nine studies that evaluated expression levels of exosomal miRNAs in GC resistance (Table 4). Emerging evidence highlights exosomal miR‐493 as a critical mediator of chemoresistance in GC with peritoneal metastasis, particularly in response to PTX‐based regimens. To delineate its functional significance, Makinoya et al. conducted a comprehensive analysis involving 50 GC patients [79]. Exosomes were isolated from peritoneal lavage fluid collected during staging laparoscopy in four individuals with peritoneal metastasis, all of whom received intraperitoneal and intravenous PTX in combination with an oral formulation. In parallel, tumour tissues from 45 patients with post‐gastrectomy recurrence who were treated with PTX‐based chemotherapy were examined to contextualise the clinical relevance of miR‐493 expression [79].

**TABLE 4:** Exosomal ncRNAs (miRNAs, lncRNAs and circRNAs) involved in gastric cancer drug resistance.

NcRNA	Diameter size (nm)	Exosome isolation	Detection methods	Clinical patients	Sample	Drug	Expression pattern	Role in chemoresistance	Targets or pathways	Biomarker type	Mechanisms	References
miR‐493	100	Centrifugation/Precipitation	qRT‐PCR/Western Blotting	Tissue samples were from patients who experienced recurrence after gastrectomy and received PTX‐based chemotherapy	Tissue	Paclitaxel	Up	Inducing	MAD2L1 is a key component of the spindle assembly checkpoint (SAC)	Prognostic	miR‐493 suppresses MAD2L1 and impairs SAC function, induces chemoresistance to intraperitoneal paclitaxel	Makinoya et al. [79]
miR‐301b‐3p	50‐200	Ultracentrifugation/Precipitation	qRT‐PCR/Western Blotting/RIP assay	DDP/VCR‐sensitive GC tissues	Tissue	Cisplatin and Vincristine	Up	Inducing	TXNIP pathway	Predictive	EV‐miR‐301b‐3p ↑ → TXNIP ↓ → MDR ↑	Zhu et al. [80]
miR‐769‐5p	40–150	Centrifugation/Precipitation	qRT‐PCR/Western Blotting/RNA‐seq/Dual‐Luciferase Reporter Assay	Serum Collected from 19 cisplatin‐resistant and 41 Cisplatin‐sensitive/75 pairs of GC tumour and normal tissues (150 total)	Tissue/serum	Cisplatin	Up	Inducing	ubiquitination degradation of p53 pathway	Prognostic	Exosome‐transmitted miR‐769‐5p confers cisplatin resistance and progression in gastric cancer by suppressing CASP9 and promoting the ubiquitination degradation of p53	Liang et al. [81]
miR‐522	30–150	Differential centrifugation/LC–MS/MS	qRT‐PCR/Western Blotting/RNA‐seq	Tissue and plasma of GC patients	Tissue/plasam	Cisplatin/paclitaxel	Up	Inducing	ALOX15 → reduction of lipid‐ROS → inhibition of ferroptosis	Prognostic	CAF secreted miR‐522 suppresses ferroptosis and promotes acquired chemo‐resistance in gastric cancer	Zhang et al. [49]
miR‐500a‐3p	100	Precipitation/Centrifugation	qRT‐PCR/Western Blotting	Plasma samples from stage III gastric cancer divided into chemoresistance and senetive	Tissue	Cisplatin	Up	Inducing	FBXW7/Cyclin E axis	Prognostic	miR‐500a‐3p inhibits FBXW7 enhancing cancer stem cell properties	Lin et al. [82]
miR‐223	80–120	Ultracentrifugation	qRT‐PCR/Western Blotting	Plasma and tissue from GC patients	Plasma/tissue	Doxorubicin	Up	Inducing	FBXW7/Cyclin E axis	Predictive	miR‐223 promotes doxorubicin resistance in gastric cancer by targeting FBXW7 and modulating EMT, with elevated plasma levels in resistant patients	Gao. et al. [83]
miR‐374a‐5p	40–100	Ultracentrifugation/Precipitation	qRT‐PCR/Western Blotting	Serum Samples: from (GC) (no pre‐operative therapies). 9 from GC with relapse after oxaliplatin. 5 from patients with pre‐cancerous gastric lesions. 17 from gastritis patients. 17 from healthy donors.	Serum	Oxaliplatin	Up	Inducing	Neurod1 axis	Prognostic/Predictive	Exosomal miR‐374a‐5p enhances oxaliplatin chemoresistance in gastric cancer by targeting Neurod1, upregulating multidrug resistance proteins, and reducing cell apoptosis.	Ji et al. [84]
LncRNA DACT3‐AS1	30–150	Ultracentrifugation	qRT‐PCR/Western Blotting/RNA‐seq	Post‐surgery tumour sample	Tissue	Oxaliplatin	Down	Inducing	miR‐181a‐5p and SIRT1 pathway	Prognostic	Upregulated DACT3‐AS1 suppresses miR‐181a‐5p, increasing SIRT1 expression, which promotes ferroptosis and inhibits EMT, ultimately enhancing oxaliplatin sensitivity	Qu et al. [27]
LncRNA CRNDE	50–150	Precipitation	qRT‐PCR/Western Blotting/RNA‐seq/	Serum and tissues from GC patients who underwent curative resection	Serum/tissue	Cisplatin	Up	Inducing	PTEN axis/PI3K/Akt pathway	Prognostic	LncRNA CRNDE, delivered by M2 macrophage‐derived exosomes, enhances cisplatin resistance in gastric cancer by promoting NEDD4‐1‐mediated PTEN degradation and activating the PI3K/Akt pathway	Xin et al. [85]
LncRNA HOTTIP	50–150	Ultracentrifugation/Precipitation	qRT‐PCR/Western Blotting	Serum samples from GC patients receiving cisplatin treatment	Serum	Cisplatin	Up	Inducing	miR‐218/HMGA1 axis	Predictive	Exosomal lncRNA HOTTIP, elevated in cisplatin‐resistant gastric cancer cells, enhances resistance by sponging miR‐218 to upregulate HMGA1, while its knockdown restores sensitivity, and high serum levels in resistant patients indicate potential as a diagnostic and prognostic biomarker.	Wang. et al. [86]
CircTEX2	42–197	Precipitation	qRT‐PCR/Western Blotting	Peripheral blood samples from Gastric patients underwent primary resection	Plasma	Cisplatin	Up	Inducing	ABCC1 and MRP1 pathway	Predictive	CircTEX2 functions as a microRNA sponge for miR‐145 → Upregulates ABCC1 encodes MRP1 → Cisplatin resistance	Qu et al. [27]
CircPVT1	100	Precipitation/Ultracentrifugation	qRT‐PCR/Western Blotting/Dual‐Luciferase Reporter Assay	Blood samples collected from patients received cisplatin therapy	Serum	Cisplatin	Up	Inducing	miR‐30a‐5p/YAP1 axis	Predictive	circ‐PVT1 suppress miR‐149‐5p then increasing in YAP1 via modulating autophagy, invasion and apoptosis	Yao et al. [87]
CircLDLRAD3	50–100	Differential centrifugation/exoEasy Maxi Kit	qRT‐PCR/Western Blotting/RNA‐seq/Dual‐Luciferase Reporter Assay	Tumour and normal tissue samples were surgically excised post‐DDP chemotherapy.	Tissue	Cisplatin	Up	Inducing	inhibits miR‐588 then increasing SOX5 pathway	Predictive	circ‐LDLRAD3 inhibits miR‐588 then increasing SOX5 expression and resulting in DDP resistance	Liang et al. [81]
CircHIPK3	100	Ultracentrifugation	qRT‐PCR/Western Blotting/Dual‐Luciferase Reporter Assay	Serum samples and tissues from divided into cisplatin resistance and senetive	Serum/Tissue	Cisplatin	Up	Inducing	circHIPK3/miR‐508‐3p/Bcl‐2/beclin1/SLC7A11 axis	Predictive	circHIPK3 expression drives cisplatin resistance in gastric cancer by inhibiting autophagy‐dependent ferroptosis, while its knockdown enhances chemosensitivity through the circHIPK3/miR‐508‐3p/Bcl‐2/beclin1/SLC7A11 axis	Shang et al. [88]
Circ0063526	120	Precipitation	qRT‐PCR/Western Blotting/RNA‐seq/Dual‐Luciferase Reporter Assay	Tissue samples and serum samples from resistance and responced groups	Tissue/serum	Cisplatin	Up	Inducing	miR‐449a/SHMT2 axis	Predictive	circ_0063526, which sponges miR‐449a, upregulating SHMT2 and altering metabolism and survival pathways, thereby increasing cisplatin resistance in gastric cancer.	Yang et al. [89]
CircPRRX1	50–80	Precipitation	qRT‐PCR/Western Blotting/RNA‐seq/	Fresh primary gastric tumour tissues and paired noncancerous	Tissue	Radiation	Up	Inducing	miR‐596/NFkB axis	Predictive	circPRRX1 sponges miR‐596, upregulating NF‐κB activating protein and increasing MMP2 and MMP9, promoting proliferation, migration, and invasion while reducing radiation sensitivity in gastric cancer.	He et al. [90]
CircPRRX1	NA	Ultracentrifugation	qRT‐PCR/Western Blotting/Dual‐Luciferase Reporter Assay	Serum from GC patients who received doxorubicin treatment	Serum	Doxorubicin	Up	Inducing	MiR‐3064‐5p/PTPN14 Signalling	Predictive	circPRRX1 drives doxorubicin resistance in gastric cancer by sponging miR‐3064‐5p, upregulating PTPN14, and enhancing malignancy	Yang et al. [55]

The study demonstrated that exosomal miR‐493 present in peritoneal fluid actively drives PTX resistance in GC by suppressing MAD2L1 expression. Comprehensive miRNA profiling identified miR‐493 as markedly upregulated in the CY^+^ group (*p* = 0.002), among six miRNAs that were differentially expressed in association with chemoresistance [79].

Exosomal miR‐301b‐3p has been identified as a key regulator of multidrug resistance in GC, primarily through the repression of thioredoxin‐interacting protein (TXNIP). Zhu et al. [80] investigated this mechanism in a cohort of 100 GC patients. The cohort included patients with CDDP‐resistant tumours (*n* = 32), vincristine (VCR)‐resistant tumours (*n* = 28) and drug‐sensitive tumours (*n* = 40), as well as 43 matched adjacent normal tissues [80].

The study revealed that MSC‐derived exosomal miR‐301b‐3p significantly enhances resistance to both CDDP and VCR by downregulating TXNIP. Elevated levels of miR‐301b‐3p were consistently observed in drug‐resistant tissues (*p* < 0.05). A significant inverse correlation in expression levels between miR‐301b‐3p and TXNIP was noted in resistant tissues (*p* < 0.05) [80]. Collectively, these findings identify exosomal miR‐301b‐3p as a pivotal effector of multidrug resistance in GC via TXNIP suppression, underscoring its potential as a mechanistically validated biomarker and potential therapeutic target.

The role of exosome‐derived miR‐769‐5p in driving CDDP resistance in GC was uncovered by Jing et al., who analysed serum samples from 60 GC patients (19 CDDP‐resistant and 41 CDDP‐sensitive) and 75 paired tumour‐normal tissues [100].

The findings spotlight exosomal miR‐769‐5p as a key regulator of CDDP resistance, specifically targeting CASP9 and promoting the ubiquitination and degradation of p53. This research underscores the role of exosomal miR‐769‐5p as a significant contributor to CDDP resistance in GC by modulating CASP9 and p53, indicating its potential as both a biomarker and a therapeutic target.

Zhang et al. [49] unveiled the pivotal role of exosome‐derived miR‐522 in promoting chemoresistance in tumour tissues and plasma samples from GC patients by inhibiting ferroptosis [101]. The investigation revealed that CAF‐derived exosomal miR‐522 promotes resistance to CDDP and PTX by targeting ALOX15, a crucial mediator of ferroptosis.

miR‐223, transported by macrophage‐derived exosomes, has emerged as a crucial factor contributing to Dox resistance in GC, as intricately demonstrated by Gao et al. The study utilised plasma samples from 30 GC patients and 20 healthy controls, with patients classified as doxorubicin‐resistant or sensitive based on relapse occurring within or beyond 6 months post‐therapy [83]. GC tissues were collected before treatment. Plasma exosomal miR‐223 levels were significantly higher in GC patients compared to controls, with elevated levels observed in doxorubicin‐resistant patients and those experiencing relapse (*p* < 0.05).

miR‐374a‐5p has garnered attention as a significant contributor to oxaliplatin resistance in GC. In a comprehensive study involving 59 GC patients, Ji et al. [84] examined its effects using patient serum, integrating molecular insights with clinical relevance [84]. Serum profiling across diverse cohorts—including GC patients who experienced relapse after oxaliplatin treatment, individuals with precancerous lesions and healthy controls—revealed that elevated levels of miR‐374a‐5p are a consistent feature in resistant cases. Consequently, this study reinforces the role of miR‐374a‐5p as a molecular switch for oxaliplatin resistance.

The TME imparts an additional layer of complexity to chemoresistance, largely through immune cell‐derived extracellular vesicles. For instance, Dong et al. [102] elucidated the role of exosomes from tumour‐associated neutrophils (TANs) in conferring chemoresistance in GC [102]. They demonstrated that N2‐polarised TANs release exosomes enriched with miR‐9‐3p. Upon internalisation by GC cells, this exosomal miRNA directly targets and downregulates Acyl‐CoA Synthetase Long‐Chain Family Member 4 (ACSL4), a key regulator of ferroptosis. This suppression of ACSL4 inhibits ferroptosis, an iron‐dependent form of cell death, thereby shielding cancer cells from oxaliplatin‐induced cytotoxicity. This mechanism was validated both in vitro and in vivo, as exosome administration counteracted the therapeutic efficacy of oxaliplatin in murine models. Collectively, these findings highlight a novel pathway wherein TANs modulate chemotherapeutic response via the exosomal miR‐9‐3p/ACSL4 axis, presenting a potential therapeutic target to overcome drug resistance in GC.

Similarly, the interplay between tumour‐associated macrophages (TAMs) and GC cells constitutes another critical axis of chemoresistance, as elucidated by another investigation [103]. This study identified that M2‐polarised TAMs secrete exosomes laden with miR‐1911–5p, which are subsequently internalised by GC cells. The transfer of this exosomal miRNA induces cisplatin resistance through the inhibition of ferroptosis. Mechanistically, miR‐1911–5p directly targets and suppresses the transcription factor MYB. The resultant downregulation of MYB, in turn, modulates the expression of AKR1B10 and its interaction with Acetyl‐CoA Carboxylase (ACC), which are key mediators of lipid metabolism and ferroptosis regulation. Consequently, this exo‐miR‐1911–5p/MYB/AKR1B10/ACC signalling cascade effectively shields cancer cells from cisplatin‐induced ferroptotic death. Furthermore, the study unveiled a reciprocal feedback loop wherein GC cells promote the M2 polarisation of TAMs, highlighting a complex crosstalk mediated by exo‐miR‐1911–5p that reinforces an immunosuppressive and chemoresistant TME.

##### 3.2.1.5 Oesophageal Squamous‐Cell Carcinoma

According to our systematic review, only two studies focused on the role of exosomal miRNAs in patients with resistance to ESCC (Table 5). Zhao et al. investigated the role of exosome‐derived miR‐21 in CDDP resistance in ESCC, utilising a cohort of 45 patients who underwent four cycles of CDDP‐based chemotherapy following surgery [91]. Tumour tissues, adjacent non‐cancerous tissues and peripheral blood were analysed, with clinicopathological features assessed using the TNM staging system. The study demonstrated that CAF‐derived exosomal miR‐21 significantly enhances CDDP resistance. Levels of exosomal miR‐21 in peripheral blood showed a positive correlation coefficient (*r*) with the proportions of monocytic myeloid‐derived suppressor cells (M‐MDSCs) (*r* = 0.593, *p* = 0.035), while miR‐21 expression in tumour tissue correlated with CAF (α‐SMA; *r* = 0.354, *p* = 0.035) and M‐MDSC (CD11b; *r* = 0.327, *p* = 0.03) markers [91]. Clinically, patients exhibiting CDDP resistance showed higher levels of exosomal miR‐21 and interleukin‐6 (IL‐6) in both blood and tumours (*p* < 0.05). Additionally, elevated expression of α‐SMA and CD11b, which correlated with miR‐21, was associated with metastasis (*p* < 0.001) [91].

**TABLE 5:** Exosomal ncRNAs (miRNAs, lncRNAs, and circRNAs) involved in oesophageal cancer drug resistance.

NcRNA	Diameter size (nm)	Exosome isolation	Detection methods	Clinical patients	Sample	Drug	Expression Pattern	Role in Chemoresistance	Targets or Pathways	Biomarker Type	Mechanisms	References
miR‐21	30–150	Ultracentrifuge	qRT‐PCR/Western Blotting/RNA‐seq	Tumour tissues after surgical treatment/Peripheral blood from patients with ESCC after four cycles of DDP	Tissue/Peripheral blood	Cisplatin	Up	Inducing	PTEN/STAT3/Monocytes to M‐MDSCs	Prognostic	Downregulating the PTEN and activating STAT3	Zhao et al. [91]
miR‐143‐3p	50–150	Ultracentrifuge	qRT‐PCR/Western Blotting/RNA‐seq	Serum from patients with ESCC with definitive radiotherapy (pre‐radiotherapy (baseline) and during radiotherapy (20–23 fractions))	Serum	Radiation	Up	Inducing	Internalised by macrophages → Induces M2 polarisation	Prognostic	Immune response and macrophage polarisation	Gao et al. [92]
LncRNA MIAT	30–100	Ultracentrifugation/Percipitation	qRT‐PCR/Western Blotting/RNA‐seq/ChIP	Serum sample after PTX treatment and resection of ESCC	Serum	Paclitaxel	Up	Inducing	TAF1/SREBF1 axis	Predictive	Activating the TAF1/SREBF1 axis	Zhang et al. [93]
LncRNA POU3F3	20–1200	Ultracentrifugation/Precipitation	qRT‐PCR/Western Blotting/ELISA	Blood samples from patients with locally advanced ESCC treated with CCRT	Plasma	Cisplatin	Up	Inducing	IL‐6 secretion	Prognostic	NF activation into CAFs → Increased IL‐6 secretion	Tong et al. [94]
Circ0000337	40–100	Ultracentrifugation	qRT‐PCR/Western Blotting/RNA‐seq/Dual‐Luciferase Reporter Assay	Tissue sample after CDDP treatment	Tissue	Cisplatin	Up	Inducing	JaK2 pathway	Prognostic	↑JaK2 expression via miR‐377‐3p sponge	Zang et al. [95]

In the evolving landscape of treatment resistance in ESCC, exosomal miR‐143‐3p has emerged as a critical modulator of radiotherapy failure, acting through the immune reprogramming of the TME. Gao et al. provided compelling evidence from a longitudinal cohort of 46 patients with locally advanced ESCC undergoing definitive radiotherapy (median dose: 60.62 Gy), with serial serum samples analysed at 80 time points throughout the treatment [92]. A subset of 21 patients also received concurrent chemotherapy [92].

Initial miRNA profiling of six representative patients (three with disease progression and three progression‐free) revealed miR‐143‐3p as one of the most prominently upregulated transcripts in resistant cases (*p* = 0.038). This finding was subsequently validated in the full cohort [92]. Collectively, these results position exosomal miR‐143‐3p as a central coordinator of radiation resistance in ESCC, with strong clinical correlations and mechanistic specificity that highlight its potential as both a biomarker and an immunomodulatory therapeutic target.

#### 3.2.2 Drug Resistance Mediated by Exosomal lncRNAs in GI Cancers

##### 3.2.2.1 Colorectal Cancer

Several exosome‐derived lncRNAs have been implicated in mediating resistance to chemotherapeutic agents in CRC, exhibiting distinct patterns based on the type of drug used (Table 1). In total, eight studies were identified that investigate the role of lncRNAs in drug resistance. Notably, among these, six lncRNAs have been associated with resistance to oxaliplatin: FAL1, CACClnc, PGM5‐AS1, CCAL and H19. For instance, the increased expression of the CAF‐derived lncRNA FAL1 (*p* < 0.001) in tissue samples from 98 CRC patients enhanced oxaliplatin resistance by promoting TRIM3‐mediated polyubiquitination of Beclin1, thereby suppressing autophagic cell death [48]. Similarly, Ren et al. found that lncRNA H19, also derived from CAFs in tissue samples from 10 patients, was significantly upregulated (*p* < 0.05) and conferred oxaliplatin resistance by activating β‐catenin signalling, which reduces treatment sensitivity [52]. In contrast, lncRNA PGM5‐AS1 was found to be downregulated (*p* < 0.01) in tissue samples from 62 patients with resistant cells. Its restoration through engineered exosomes reversed oxaliplatin resistance by modulating alternative splicing and the expression of NME1 and PAEP [50]. Plasma‐derived CACClnc was examined in 34 patients by Zhang et al. [49]. This lncRNA was found to be upregulated (*p* < 0.05) and promoted resistance by modulating the alternative splicing of RAD51. Similarly, CCAL (*p* < 0.05), identified in tissue samples from 30 patients, mediated oxaliplatin resistance through activating β‐catenin signalling [51]. In a study involving 20 CRC patients, Jun‐Hong Hu et al. found that LINC00355 was significantly upregulated (*p* < 0.01), while its downstream target, miR‐34b‐5p, was downregulated (*p* < 0.01) [53]. This regulatory imbalance contributed to resistance against 5‐FU, oxaliplatin and CDDP by upregulating CRKL and inducing EMT. Additionally, two other lncRNAs—HOTTIP and UCA1—were associated with resistance to mitomycin and cetuximab, respectively. HOTTIP, which was upregulated in both serum (*p* < 0.05) and tissue samples (*p* < 0.05) from 95 CRC patients, was found to disrupt the miR‐214‐mediated degradation of KPNA3 [104]. In contrast, UCA1, identified in serum‐derived exosomes from 53 patients, was also upregulated (*p* < 0.01) and linked to cetuximab resistance, although its mechanism of action remains unclear [54]. Overall, the interaction between exosomal lncRNAs and drug response in CRC highlights a complex network of resistance mechanisms that vary according to therapeutic agents and regulatory pathways.

##### 3.2.2.2 Pancreatic Ductal Adenocarcinoma

Only one study evaluated the role of exosomal lncRNAs in patients with resistance to PDAC (Table 2). In this clinical study conducted, Chi et al. examined tissue samples from 35 pancreatic cancer cases, comprising 23 male and 12 female participants. All received GEM treatment. This research revealed that lncRNA UCA1 was upregulated in PC tissues (*p* < 0.05) [69]. Additionally, the study demonstrated that under low oxygen conditions, exosomes released by pancreatic stellate cells (PSCs) delivered lncRNA UCA1 to pancreatic cancer cells. Within the nucleus of these cells, lncRNA UCA1 recruited EZH2 to the SOCS3 promoter, increasing histone methylation and suppressing SOCS3 transcription. This mechanism eventually increases the tolerance of cancer cells to GEM. In addition, increased expression of lncRNA UCA1 can reduce expression of miR‐124 (*p* < 0.05) [69].

##### 3.2.2.3 Hepatocellular Carcinoma

To date, only a single study has assessed the function of exosomal lncRNAs in patients with treatment‐resistant HCC (Table 2). Mingbo Cao et al. in a study on 55 patients from China exhibited that exosome‐derived lnc‐FAM72D‐3 overexpressed (*p* < 0.05) in tissue and serum and linked with lenvatinib resistance (LR). Moreover, this study revealed overexpression of lnc‐FAM72D‐3 downregulates the expression levels of the MBNL1 protein by promoting its ubiquitin‐mediated degradation through HECTD3. This mechanism further inhibits the interaction between MBNL1 and FAK, resulting in the nuclear exclusion of FAK and its subsequent phosphorylation. Finally, this cascade activates downstream signalling pathways, including PI3K/AKT/mTOR and β‐catenin/c‐myc, along with signalling molecules such as Paxillin, which leads to the remodelling of the cytoskeleton in HCC cells and fosters malignant behaviours. This events thereby promotes the progression of LR [105].

##### 3.2.2.4 Gastric Cancer

Within the complex interplay of tumour‐stroma communication in GC, the lncRNA DACT3‐AS1 has emerged as a pivotal regulatory node in modulating oxaliplatin resistance (Table 4). Qu et al. delineated this role through a multi‐level investigation involving 93 clinical GC specimens and 25 tumour samples stratified by oxaliplatin sensitivity [27]. DACT3‐AS1 was consistently downregulated in GC tissues compared to their non‐malignant counterparts (*p* < 0.05), with its loss correlating with enhanced malignancy and chemoresistance. The upregulation of miR‐181a‐5p in resistant samples correlated with reduced expression of its downstream effector, SIRT1 (*p* < 0.05) [27]. Overall, these findings highlight the loss of CAF‐derived exosomal DACT3‐AS1 as a driver of oxaliplatin resistance in GC, presenting compelling translational potential as a dual‐function biomarker and therapeutic target within the chemoresistance landscape of GC.

In the landscape of drug resistance in GC, lncRNA CRNDE emerged as a pivotal force, meticulously dissected by Xin et al. (Table 4) [85]. Their investigation explored the molecular mechanisms underpinning CDDP resistance in GC patients. The study draws upon 35 pairs of cancerous and adjacent normal tissues, alongside peripheral blood monocytes from healthy volunteers [85].

Exosomal CRNDE, which is enriched in tumour‐associated macrophages (TAMs), plays a pivotal role in driving CDDP resistance. Notably, CRNDE was significantly upregulated in GC tissues and exhibited a strong positive correlation with CD63 + CD163+ M2 macrophages (*r* = 0.8434, *p* < 0.05). This upregulation was not limited to the tumour tissue; elevated levels of CRNDE were also observed in TAMs and patient serum samples [85].

lncRNA HOTTIP has emerged as a potent orchestrator of CDDP resistance in GC, as masterfully explored by Wang et al. [86] (Table 4). The study harnessed serum samples from 58 GC patients who were treated with CDDP. The patients were categorised into responder (*n* = 30) and non‐responder (*n* = 28) groups according to the RECIST 1.1 criteria [86]. Serum exosomal HOTTIP levels were found to be higher in non‐responders and remained stable across different conditions, demonstrating diagnostic potential (AUC = 0.743, *p* < 0.05) [86]. These results establish exosomal HOTTIP as a critical mediator of CDDP resistance in GC, offering robust clinical evidence for its potential as a diagnostic biomarker and therapeutic target.

##### 3.2.2.5 Oesophageal Squamous‐Cell Carcinoma

Limited studies have evaluated the role of exosomal lncRNAs in patients with resistance to ESCC (Table 5). Lin et al. examined the role of exosome‐derived lncRNA MIAT in PTX resistance in ESCC by studying 48 patients who received PTX treatment post‐resection [93]. Serum samples were collected before chemotherapy. Exosomal MIAT was significantly upregulated in non‐responders (*n* = 28) compared to responders (*n* = 20) (*p* < 0.05), indicating its diagnostic potential for PTX resistance (AUC = 0.728, 95% CI: 0.559–0.898) [93]. These findings underscore exosomal MIAT as a mediator of PTX resistance in ESCC, with clinical and diagnostic relevance (AUC = 0.728).

In the context of CDDP resistance in ESCC, exosomal lncRNA POU3F3 has emerged as a key mediator in reshaping the TME. Tong et al. dissected its role by examining 138 patients with locally advanced ESCC who were undergoing concurrent chemoradiotherapy, along with plasma samples from 78 patients with postoperative recurrence [94]. The study revealed that exosomal lncRNA POU3F3 significantly promotes CDDP resistance by triggering fibroblast differentiation into CAFs, thereby altering the TME [94].

In ESCC, the heterogeneity of CAFs has been identified as a crucial factor in therapeutic resistance. A study by Zhou et al. demonstrated that a specific subpopulation of CAFs, characterised by the expression of Fibroblast Activation Protein (FAP^+^CAFs), significantly promotes tumour progression and radioresistance [106]. The investigation revealed that FAP expression in the tumour stroma was positively correlated with aggressive clinical features, including lymph node metastasis and poor patient survival. Mechanistically, FAP+ CAFs were found to secrete exosomes containing the long ncRNA AFAP1‐AS1. This exosomal lncRNA is transferred to ESCC cells, where it enhances their resistance to radiation by promoting more efficient DNA damage repair. This effect was confirmed both in vitro, where exosomes from FAP+ CAFs increased the radioresistance of cancer cells and in vivo, where co‐injection of FAP+ CAFs with tumour cells led to accelerated tumour growth following irradiation. Furthermore, the study highlighted the clinical relevance of this axis, showing that elevated plasma levels of AFAP1‐AS1 in ESCC patients correlated with a poor response to chemoradiotherapy [106]. These findings pinpoint the transfer of exosomal AFAP1‐AS1 from FAP+ CAFs as a key mechanism driving radioresistance in ESCC.

#### 3.2.3 Drug Resistance Mediated by Exosomal circRNAs in GI Cancers

##### 3.2.3.1 Colorectal Cancer

Five circRNAs have been associated with 5‐FU resistance in CRC (Table 1). Yang et al. reported that circ_0067557, which is upregulated (*p* < 0.01) in exosomes derived from CAFs of 12 patients, promotes resistance to 5‐FU and oxaliplatin by enhancing the expression of Lin28A and Lin28B [55]. Furthermore, the upregulation of circ_0000338 in tissue samples (*p* < 0.05) from 60 patients and serum samples (*p* < 0.01) from 17 patients enhances 5‐FU resistance by downregulating miR‐217 and miR‐485‐3p [57, 60]. Similarly, in a cohort of 60 patients, the upregulation of circ‐0004771, detected in both serum (*p* < 0.05) and tissue samples (*p* < 0.05), increases 5‐FU resistance via the miR‐653/ZEB2 signalling pathway [61]. In contrast, circ_0094343 is downregulated (*p* < 005) in tissue samples from 20 patients with resistant cells. Its expression sensitises CRC cells to 5‐FU by regulating glycolysis through the miR‐766‐5p/TRIM67 axis [64].

Resistance to oxaliplatin has been associated with seven distinct circRNAs (Table 1). Among these, circ_0067557 and circ_0094343 that it suggested previously implicated in 5‐FU resistance [55, 64], also contribute to oxaliplatin resistance, underscoring their broader involvement in chemoresistance. Additionally, Deng et al. [56] identified circ_0001610, which is significantly upregulated in serum (*p* < 0.05) and contributed to oxaliplatin resistance by activating PGC‐1α‐dependent oxidative phosphorylation (OXPHOS). In a study conducted by Wang et al. [107], circ_0005963 was found to be significantly upregulated in serum (*p* < 0.01), while its target, miR‐122, was downregulated (*p* < 0.05). This upregulation promotes glycolysis by regulating PKM2 via the suppression of miR‐122 in serum samples from 19 patients. Moreover, Pan et al. [62] discovered that circATG4B, which is upregulated in tumour tissues (*p* < 0.05), induces resistance by promoting autophagy and preventing TMED10 from binding to ATG4B. Additionally, upregulation of circN4BP2L2 (*p* < 0.01), identified by Zhan Qu et al. [63] in tissues from 10 CRC patients, promotes chemoresistance by activating the PI3K/AKT/mTOR signalling pathway through its interaction with EIF4A3. Xu et al. [108] reported that circ‐FBXW7 was significantly downregulated (*p* < 0.05) in tumour tissues from 56 oxaliplatin‐resistant CRC patients. Its low expression was associated with enhanced activity of miR‐18b‐5p, increased EMT, reduced apoptosis and poor treatment response. Regarding doxorubicin resistance, Zhang et al. [109] demonstrated that circ_0006174 was upregulated (*p* < 0.001) and functioned by sponging miR‐1205, which led to the upregulation of CCND2 expression. This mechanism reduced CRC tissue samples, sensitivity from 41 patients.

##### 3.2.3.2 Pancreatic Ductal Adenocarcinoma

Multiple studies have established a strong correlation between dysregulated exosomal circRNA profiles and acquired resistance to chemotherapeutic agents in PDAC (Table 2). In this context, Zeng et al. conducted a study involving 40 Chinese patients who were treated with GEM, during which both tissue and blood specimens were collected for analysis. The study revealed that elevated levels of circZNF91 (*p* < 0.01) enhanced circZNF91 production in PC cells independently of ZNF91 mRNA levels, while significantly increasing resistance to GEM treatment [70]. CircZNF91 contains multiple Ago2 binding sites and numerous miRNA targets, with miR‐23b‐3p showing particularly strong interaction potential through its 24 binding sites. Functionally, circZNF91 acts as an effective molecular sponge for miR‐23b‐3p, with experimental evidence showing that the overexpression of circZNF91 leads to a significant reduction in miR‐23b‐3p levels (*p* < 0.01). Notably, miR‐23b‐3p has been identified as a robust inhibitor of GEM resistance in PDAC cells, whose effect could be completely rescinded by reversing the upregulation of circZNF91 [70]. SIRT1 stabilises HIF‐1α protein via deacetylation, and the circZNF91/miR‐23b‐3p/SIRT1 axis serves as a critical regulatory pathway for HIF‐1α protein maintenance. Meanwhile, this investigation revealed that elevated circZNF91 levels, along with hypoxic exosomes, potently enhance the stability of HIF‐1α protein. By stabilising HIF‐1α and subsequently activating glycolytic pathways, circZNF91 drives GEM resistance in PC cells. Furthermore, hypoxic conditions may stimulate PC cells to release exosomes enriched with circZNF91. These exosomes could disseminate to normoxic regions, facilitating the transition of normoxic PC cells to a hypoxic phenotype by enhancing glycolytic metabolism and promoting chemotherapeutic resistance [70].

##### 3.2.3.3 Hepatocellular Carcinoma

Several studies have examined the role of circRNAs in HCC development, particularly in progression and drug resistance (Table 3). For instance, Gong et al. investigated 64 tissue samples from patients with HCC and demonstrated that the up‐regulation of circDCAF8 (*p* < 0.001) promotes proliferation, metastasis, angiogenesis and resistance to Regorafenib [75]. Furthermore, circDCAF8 acts as a sponge for miR‐217, and its overexpression leads to a reduction in miR‐217 levels. NAP1L1 has been identified as a target gene of miR‐217, and decreased miR‐217 activity has been shown to result in elevated NAP1L1 mRNA expression. This reciprocal relationship was confirmed by the impressive repression of NAP1L1 following the treatment of miR‐217 mimics. As noted above, circDCAF8 promotes HCC progression through regulation of the miR‐217/NAP1L1 axis [75]. The exosomal circUPF2 has recently been identified as a mediator of sorafenib resistance in HCC. Clinical evidence from a case control study conducted by Dong and colleagues, which analysed tissues and blood samples from 36 patients (31 male and 5 female) and 20 healthy controls, showed that elevated circUPF2 expression (*p* < 0.001) is associated with enhanced chemoresistance to sorafenib [76]. Moreover, their investigation revealed that exosome‐mediated sorafenib resistance in HCC cells was linked to reduced intracellular lipid reactive oxygen species (ROS) and Fe^2+^ concentrations, along with elevated glutathione reflecting the inhibition of ferroptosis [76]. On this topic, a separate study by Hu et al. involving 58 patients exhibited that circCCAR1 was upregulated in HCC tissues and blood (*p* < 0.001) and was responsible for improved resistance to anti‐PD1 immunotherapy [77]. Two crucial key factors, namely E1A binding protein p300 (EP300) and eukaryotic translation initiation factor 4A3 (EIF4A3), enhanced the biogenesis of circCCAR1 by increasing cyclisation and nuclear export of circCCAR1. Moreover, Wilms tumour 1‐associated protein (WTAP)‐mediated m6A modification can affect the circCCAR1 stability through a combination with insulin‐like growth factor 2 mRNA‐binding protein 3 (IGF2BP3). On the other hand, circCCAR1 functions as a sponge for miR‐127‐5p, thereby upregulating its target, WTAP. This interaction establishes a feedback loop via the circCCAR1/miR‐127‐5p/WTAP axis [77]. An experimental study involving 55 tissue and blood samples from HCC patients demonstrated that elevated expression of circRNA‐G004213 during transarterial chemoembolisation (TACE) enhances sensitivity to CDDP, consequently suppressing tumour progression and improving patient survival. Additionally, the study found that circ‐G004213 modulates CDDP responsiveness by targeting the miR‐513b‐5p/PRPF39 axis [78].

##### 3.2.3.4 Gastric Cancer

Multiple circRNAs have been identified and linked to resistance to chemotherapy in GC (Table 4). A study conducted by Qu et al. investigated the role of exosome‐derived circTEX2 in mediating CDDP resistance in GC. The research utilised GC cell lines (AGS, MKN45), THP‐1‐derived macrophages and peripheral blood samples from 10 GC patients [110].

The findings revealed that M2‐polarised macrophages significantly enhance CDDP resistance through exosomal circTEX2, which regulates the miR‐145/ABCC1 axis. M2 macrophages were found to reduce CDDP‐induced apoptosis and increase cell viability (*p* < 0.01). CircTEX2 was significantly upregulated in M2 macrophages and their exosomes (*p* < 0.01), exhibiting a cytoplasmic localisation. Mechanistically, circTEX2 was found to act as a sponge for miR‐145, leading to the upregulation of ABCC1 expression (*p* < 0.01) [110].

In vivo studies demonstrated that M2‐derived exosomes containing circTEX2 promoted CDDP resistance in AGS xenografts, whereas circTEX2‐silenced exosomes did not exhibit this effect (*p* < 0.01). Additionally, human monocyte‐derived M2 macrophages from GC patients showed elevated expression of circTEX2. Furthermore, M2 exosomes were found to enhance resistance, an effect that was reversed by circTEX2 silencing (*p* < 0.05) [110].

In recent advancements in the study of chemoresistance in GC, Liang et al. identified exosome‐derived circular RNA circ‐LDLRAD3 as a pivotal regulator of CDDP resistance. The study utilised a cohort of 46 paired tumour and normal tissue samples from GC patients, categorised into CDDP‐resistant (*n* = 27) and CDDP‐sensitive (*n* = 19) groups following CDDP therapy [81].

Their investigation revealed that exosomal circ‐LDLRAD3 plays a critical role in promoting CDDP resistance by acting as a sponge for miR‐588, which subsequently leads to the upregulation of SOX5. Furthermore, there was a notable downregulation of cyclin D1 and MMP9 expression (*p* < 0.01) [81].

On a mechanistic level, circ‐LDLRAD3 functions as a competing endogenous RNA (ceRNA) for miR‐588, which is downregulated in resistant tissues and inversely correlated with circ‐LDLRAD3 expression (*p* < 0.05). MiR‐588 targets the 3′ untranslated region (UTR) of SOX5, leading to a reduction in its expression (*p* < 0.05). However, SOX5 expression was significantly elevated in CDDP‐resistant tissues (*p* < 0.05) [81].

Yao et al. investigated the critical role of exosome‐derived circ‐PVT1 in enhancing CDDP resistance in GC. The study analysed serum samples from 60 GC patients, comprising 30 CDDP‐resistant and 30 CDDP‐sensitive individuals [87].

The study illuminated that exosomal circ‐PVT1 contributes to CDDP resistance by sponging miR‐30a‐5p, which in turn upregulates YAP1. Exosomal circ‐PVT1 was significantly elevated in the serum of CDDP‐resistant patients (*p* < 0.05), with 50% of resistant tissues showing high expression levels. This elevation correlated with advanced tumour‐node‐metastasis grade (*p* = 0.0029), lymph node metastasis (*p* = 0.0099) and larger tumour size (*p* = 0.0281) [87]. Conversely, miR‐30a‐5p was downregulated in resistant samples, exhibiting a negative correlation with circ‐PVT1 (*r* = −0.576, *p* < 0.001). Dual‐luciferase assays confirmed the binding of circ‐PVT1 to miR‐30a‐5p, with its knockdown leading to an increase in miR‐30a‐5p expression. In contrast, the inhibition of miR‐30a‐5p reversed these effects, restoring resistance, invasion and autophagy (*p* < 0.05). MiR‐30a‐5p targeted the 3′UTR of YAP1, resulting in a reduction of its expression, while YAP1 was found to be upregulated in resistant serum samples (*p* < 0.05) and negatively correlated with miR‐30a‐5p (*r* = −0.6947, *p* < 0.001). MiR‐30a‐5p overexpression diminished resistance, invasion and autophagy, effects that were negated by YAP1 upregulation (*p* < 0.05) [87].

CircHIPK3 stands out as a potent driver of CDDP resistance in GC, as meticulously investigated by Shang et al. The study drew on serum and tissue samples from 60 GC patients, who were treated with CDDP‐based chemotherapy following gastrectomy. Patients were classified as either CDDP‐resistant or sensitive based on tumour recurrence [88].

The findings establish CircHIPK3 as a central regulator of CDDP resistance by suppressing autophagy‐dependent ferroptosis via the miR‐508‐3p/Bcl‐2/beclin1 axis. CircHIPK3 was significantly upregulated in resistant tissues (*p* < 0.05) [88].

Wang et al. uncovered exosomal circPRRX1 as a crucial mediator of Dox resistance in GC, utilising serum samples from 56 GC patients treated with doxorubicin. These patients were categorised into two groups based on their response to treatment: responders (*n* = 24) and non‐responders (*n* = 32), as per the RECIST criteria [90].

Mechanistically, circPRRX1 sponged miR‐3064‐5p, as validated by luciferase assay, with miR‐3064‐5p being significantly downregulated in resistant cells (*p* < 0.05). Inhibition of miR‐3064‐5p restored proliferation, migration, invasion and MMP9/MMP2 expression in circPRRX1‐silenced resistant cells (*p* < 0.05). MiR‐3064‐5p was found to target PTPN14, which was upregulated in resistant cells and circPRRX1 knockdown led to a reduction in PTPN14 expression, an effect countered by miR‐3064‐5p inhibition (*p* < 0.05). Overexpression of PTPN14 in resistant cells reversed the suppressive effects of circPRRX1 silencing on proliferation and invasion (*p* < 0.05) [90].

In clinical samples, exosomal circPRRX1 levels were significantly higher in non‐responders and remained stable under various conditions, demonstrating its potential as a diagnostic biomarker (*p* < 0.05) [90]. Another key instigator of CDDP resistance, circ_0063526, was studied by Yang et al. in GC patients. The study utilised 53 GC tissue samples, paired non‐tumour tissues and serum samples from patients, categorised into CDDP‐responsive (*n* = 26) and non‐responsive (*n* = 27) groups [89].

Circ_0063526 sponged miR‐449a, which was downregulated in GC tissues (*p* < 0.05), with binding confirmed by luciferase and RNA pull‐down assays (*p* < 0.05). Exosome treatment reduced miR‐449a, which was restored by Circ_0063526 knockdown (*p* < 0.05). miR‐449a targeted SHMT2, upregulated in GC tissues (*p* < 0.05), with luciferase and pull‐down assays validating binding (*p* < 0.05) [89].

Circ_0063526 knockdown decreased SHMT2, countered by miR‐449a inhibition (*p* < 0.05). Exosomal circ_0063526 knockdown suppressed SHMT2, migration, invasion and autophagy, reversed by SHMT2 overexpression (*p* < 0.05). Serum exosomal circ_0063526 was higher in non‐responders, stable under RNase A, NaOH and HCl, with a higher non‐response proportion in the high‐expression group (*p* < 0.05) [89].

CircPRRX1 emerges as a dynamic regulator of radiation resistance in GC, as skillfully elucidated by He et al. The study utilised 25 primary GC tumours and paired noncancerous tissues from chemonaive patients [111].

The findings reveal exosomal circPRRX1 as a promoter of proliferation, migration, invasion and radiation resistance via the miR‐596/NKAP axis. CircPRRX1 was significantly upregulated in primary gastric tumours compared to the adjacent normal gastric tissues (*p* < 0.05), with its closed‐loop structure confirmed by RNase R resistance. CircPRRX1 sponged miR‐596, downregulated in primary gastric tumours (*p* < 0.05), with binding validated by luciferase assays and inversely correlated expression (*p* < 0.05) [111].

The miR‐596 inhibition reversed the effects of si‐circPRRX1‐exo on proliferation, migration, invasion, MMP9/MMP2 and radiation sensitivity (*p* < 0.05). miR‐596 targeted NKAP, upregulated in primary gastric tumours (*p* < 0.05), with luciferase assays confirming binding and miR‐596 reducing NKAP expression (*p* < 0.05). NKAP overexpression countered miR‐596's suppressive effects on proliferation, migration, invasion, MMP9/MMP2 and radiation sensitivity (*p* < 0.05). Si‐circPRRX1‐exo reduced NKAP, reversed by miR‐596 inhibition, with NKAP positively correlated with circPRRX1 (*p* < 0.05) [111].

In a study by Zang et al. [112], a novel exosomal circRNA, named circ50547, was identified and characterised. This research demonstrated that circ50547 is highly expressed in both GC tissues and the serum exosomes of patients, with its exosomal form showing superior value as a non‐invasive diagnostic marker [112]. Functionally, increased levels of circ50547 were shown to enhance the proliferation, migration, invasion, cancer stemness and oxaliplatin resistance of GC cells. Mechanistically, the study revealed that circ50547, which is primarily located in the cytoplasm, acts as a ceRNA. It functions by sponging miR‐217, thereby preventing the miRNA from inhibiting its downstream target, Hepatocyte Nuclear Factor 1 Beta (HNF1B) [112]. This circ50547/miR‐217/HNF1B axis was shown to be a key pathway promoting the malignant progression of gastric cancer, highlighting exosomal circ50547 as a potential therapeutic target and biomarker.

##### 3.2.3.5 Oesophageal Squamous‐Cell Carcinoma

Research has demonstrated a significant link between exosomal circRNAs and reduced effectiveness of resistance chemotherapy treatment in ESCC (Table 5). For example, Zang et al. investigated exosome‐derived circ_0000337 in CDDP resistance in ESCC, analysing 52 patients (29 CDDP‐resistant, 23 CDDP‐sensitive). with characteristics including 44.23% female and 55.77% male, 26.92% aged < 60 and 73.08% ≥ 60 years and tumour locations of 15.38% upper and middle‐upper, 48.08% middle and 36.54% middle‐lower and lower. Tissue samples were collected pre‐surgery, and the experiments utilised EC9706, KYSE30 and their CDDP‐resistant variants [95].

circ_0000337 was significantly upregulated in CDDP‐resistant tissues (*p* < 0.05). Its knockdown reduced IC50, proliferation, colony formation, migration and invasion while increasing apoptosis in resistant cells (*p* < 0.05). Circ_0000337 acted as a miR‐377‐3p sponge, as validated by dual‐luciferase and biotin‐coupled miRNA capture assays (*p* < 0.05). miR‐377‐3p was downregulated in resistant tissues (*p* < 0.05) and negatively correlated with circ_0000337 (*p* < 0.05). Exosome‐mediated miR‐377‐3p suppression was reversed by miR‐377‐3p mimics, which reduced resistance (*p* < 0.05). JAK2, a miR‐377‐3p target, was upregulated in resistant tissues (*p* < 0.05), positively correlated with circ_0000337 and negatively with miR‐377‐3p (*p* < 0.05). JAK2 overexpression restored resistance in the presence of miR‐377‐3p mimics (*p* < 0.05) [95].

### 3.3 Exosomal ncRNAs as Prognostic Biomarkers in GI Cancers

**FIGURE 2:**
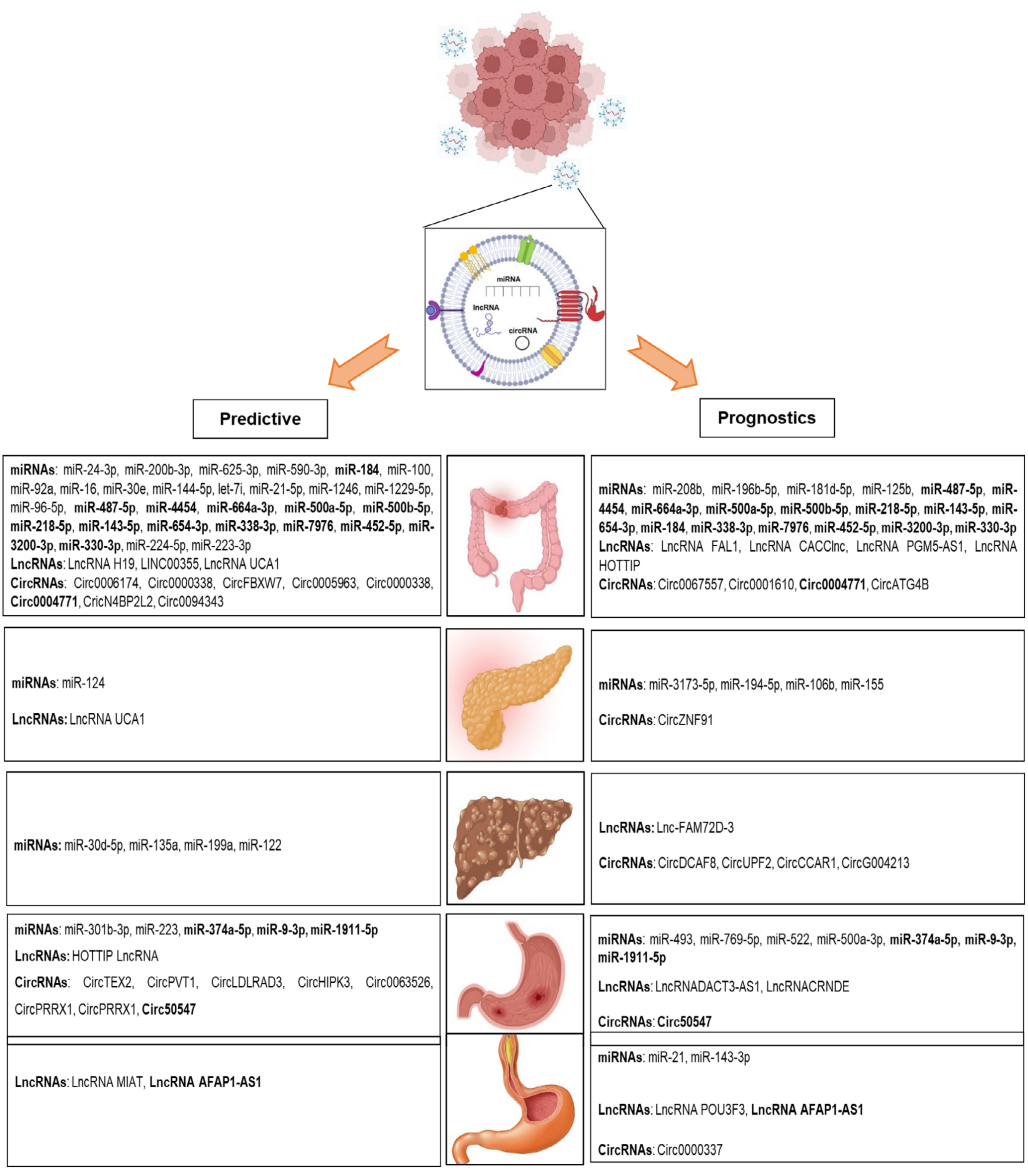
Clinical significance of exosomal non‐coding RNAs in drug resistance of gastrointestinal cancers.

This section explores the prognostic potential of exosomal ncRNAs in GI cancers. By reviewing recent studies, it highlights specific exosomal miRNAs, lncRNAs and circRNAs' association with chemoresistance, treatment outcomes and patient survival, offering promising avenues for personalised cancer management (Figure 2).

#### 3.3.1 CRC

Exosomal miRNAs play a pivotal role in informing CRC prognosis and diagnosis, offering insights into disease progression, treatment response and drug resistance. Several upregulated miRNAs have been identified in CRC patients (Table 1). For instance, exosomal miR‐24‐3p that identified in 28 patients by Zhang et al. [38], has been associated with chemoresistance via increased tumour growth under MTX treatment (*p* < 0.05), suggesting its potential as a prognostic marker. Similarly, Ning et al. [39], reported that exosomal miR‐208b, investigated in 116 cases, correlates with shorter progression‐free survival (PFS) (*p* < 0.001) and may serve as a prognostic biomarker. Other exosomal miRNAs have also demonstrated strong potential as predictive biomarkers of drug response: miR‐92a‐3p has been linked to chemoresistance in CRC cohorts [96], while miR‐196b‐5p, detected in 110 patients, has also been associated with resistance to chemotherapy (*p* = 0.006) [40]. Likewise, miR‐181d‐5p, observed in 30 CRC tissue samples, is associated with 5‐FU resistance and poor prognosis (*p* < 0.001) [43]. As showed in Table 1, miR‐590‐3p has been proposed as a predictive biomarker for radioresistance in Chinese patients [44]. In Japanese CRC patients, Yagi et al. found that elevated miR‐125b levels predicted poorer PFS (*p* < 0.01), reinforcing its value as a prognostic indicator [46]. Further supporting these findings, Guoying Jin et al. identified a panel of exosomal miRNAs (*p* < 0.01) including miR‐21–5p, miR‐1246, miR‐1229‐5p and miR‐96‐5p in a cohort of 43 Chinese patients, all of which were associated with chemoresistance [47]. In a study of 120 chemotherapy‐naive RAS/BRAF WT mCRC patients, Xu et al. developed the EXONERATE assay using a panel of 14 differentially expressed exosomal miRNAs, which robustly predicted shorter PFS, OS and lower ORR independent of tumour sidedness (*p* < 0.001), highlighting its potential as a prognostic and predictive biomarker for anti‐EGFR therapy outcomes [97]. Additionally, miR‐184, miR‐100, miR‐92a and miR‐16, were significantly dysregulated in plasma exosomes and proposed as potential predictive biomarkers for oxaliplatin resistance (*p* < 0.05) [45]. Finally, miR‐625‐3p, observed in Chinese CRC cases, may inform chemotherapy response due to its association with multidrug resistance [42]. Several downregulated exosomal miRNAs have also been implicated in CRC, particularly in relation to treatment resistance and poor prognosis. For instance, miR‐122 that detected in 19 Chinese patients by Wang et al. has been linked to chemoresistance, suggesting a potential role in predicting unfavourable treatment outcomes [107]. Similarly, miR‐200b‐3p, studied in 5 Chinese cases, has been associated with 5‐FU resistance, indicating its relevance as a prognostic marker [41]. In addition, in a study on 20 Chinese patients it indicated that miR‐34b‐5p—may inform therapeutic response due to its connection with chemoresistance [53]. miR‐653, examined in 60 cases, has shown potential in predicting chemotherapy efficacy (*p* < 0.05) [61]. Moreover, a panel consisting of miR‐30e, miR‐144‐5p and let‐7i detected in a larger cohort of 186 Chinese patients was significantly associated with oxaliplatin resistance, underscoring its potential as a predictive biomarker panel (*p* < 0.05) [45]. Additionally, low miR‐224–5p levels in 16 Chinese CRC patients are linked to 5‐FU resistance via the miR‐224–5p/S100A4 axis, with low S100A4 expression associated with better OS. Verapamil or triptolide can reverse this resistance, suggesting miR‐224–5p as a prognostic biomarker [98].

Based on Table 1, emerging evidence suggests that lncRNAs and circRNAs may serve as valuable biomarkers for the prognosis and treatment stratification of CRC, pending further clinical validation. Among the upregulated lncRNAs, FAL1 that reported by Zhu et al. in a cohort of 98 Chinese patients, was significantly associated with poorer OS rate (*p* < 0.016) and disease‐free survival (DFS) (*p* < 0.0277) [48]. Similarly, CACClnc, investigated in 34 Chinese cases, was found to predict shorter DFS (*p* = 0.027) [49]. In a study involving 30 CRC patients, Deng et al. identified CCAL as a potential prognostic marker linked to chemoresistance [51]. H19, explored in a small cohort of 10 patients, was highlighted for its role in modulating chemoresistance and influencing therapeutic response [52]. LINC00355, observed in 20 Chinese cases, demonstrated potential in identifying chemoresistant tumours [53]. Additionally, HOTTIP, evaluated in 95 patients, was found to correlate with chemotherapy response based on its expression profile [44]. UCA1, analysed by Yang et al. [54] in 53 Chinese CRC patients, emerged as a biomarker for predicting cetuximab resistance in metastatic CRC (Table 1).

Downregulated lncRNAs include PGM5‐AS1, found in 62 Chinese patients with CRC, with higher expression predicting better OS (*p* = 0.0005) [50].

Among the upregulated circRNAs, it was reported by Yang et al. [55] in 12 Chinese patients that circ_0067557 is associated with chemoresistance, suggesting its potential role as an indicator predictive of response (*p* < 0.05). The circ_0001610, detected in 25 Chinese cases, was shown to be involved in modulating treatment response [56]. In a cohort of 41 Chinese CRC patients, circ_0006174 was identified as a potential biomarker for assessing chemoresistance [109]. circ_0000338, studied across 60 Chinese and 17 Malaysian patients, emerged as a biomarker predictive of chemoresistance [57, 60]. Circ‐0004771, identified in 60 Chinese patients, serves as a predictive biomarker for treatment response (*p* < 0.05) [61]. circATG4B, examined in 128 Chinese CRC cases, was found to correlate with shorter DFS (*p* = 0.0398), indicating its potential prognostic significance [62]. circ_0005963, reported in 19 Chinese patients, may act as a prognostic marker due to its association with chemoresistance [111]. Finally, circN4BP2L2, observed in 10 Chinese patients, was proposed to inform chemotherapy response [63].

Downregulated circRNAs include circ_0094343, which Chen et al. identified in 20 Chinese patients, serving as a predictive biomarker for enhanced chemosensitivity [64].

#### 3.3.2 PDAC

As detailed in Table 2, some exosomal ncRNAs have been implicated in the prognosis of PDAC and related to tumour cell survival and resistance to standard treatments.

Ran et al. demonstrated reduced ACSL4 expression following miR‐3173–5p overexpression has been associated with decreased 5‐year survival outcomes in PDAC patients [65]. In a study by Zeng et al., PDAC patients exhibiting higher levels of circZNF91 expression were associated with reduced OS (*p* < 0.05) [70]. Jiang and colleagues showed that increased expression of miR‐194‐5p can promote the survival rate of the ALDH1A1^+^ cells [66]. Fang et al. reported that elevated miR‐106b levels in cancer cells enhance cell survival and confer resistance to GEM [67]. Kaplan–Meier analysis revealed significantly reduced OS (*p* = 0.008) and DFS (*p* = 0.021) in patients with elevated miR‐155 expression [68].

#### 3.3.3 HCC

As detailed in Table 3, specific circRNA and lncRNA have been associated with survival outcomes and drug resistance. These findings highlight circRNAs' potential utility in stratifying patients for targeted interventions.

Qin et al. showed overexpressing of the circ‐G004213 prolonged the survival rate of the HCC cells (*p* < 0.05) (*p* = 2.410 × 10^−3^) [78]. Moreover, research by Hu and colleagues demonstrated that high circCCAR1 expression in HCC patients led to decreased OS (*p* = 0.0016) and an unfavourable prognosis [77]. Additionally, the survival data indicated that higher levels of SLC7A11 were observed following the overexpression of circUPF2, which were associated with reduced OS (*p* = 0.0011) and DFS (*p* = 0.0041) in patients [76]. The survival curves showed that higher circDCAF8 levels were linked to diminished OS in individuals with HCC (*p* < 0.001) [75].

Also, survival analysis of a study by Mingbo Cao et al. indicated that HCC patients exhibiting elevated lnc‐FAM72D‐3 expression had markedly poorer OS (*p* = 0.0105) and recurrence‐free survival (RFS) (*p* = 0.0142) [105].

#### 3.3.4 GC

According to Table 4, Focusing on GC, a range of exosomal ncRNAs linked to chemoresistance and poor prognosis have been identified and demonstrate how these ncRNAs influence molecular pathways, offering new strategies for therapeutic targeting and prognosis refinement.

Increasing of exosomal miR‐493 levels is implicated in chemoresistance to intraperitoneal PTX therapy in GC patients with peritoneal metastasis, mediated through suppression of MAD2L1 expression. In a cohort of 45 recurrent GC patients treated with PTX‐based chemotherapy, low MAD2L1 expression, observed in 48.9% (22/45) of patients, was significantly associated with reduced chemosensitivity, as evidenced by a lower overall response rate in the MAD2L1‐low group compared to the MAD2L1‐high group (*p* = 0.043) [79]. Furthermore, low MAD2L1 expression correlated with significantly shorter PFS (*p* = 0.044) and a trend toward decreased OS (*p* = 0.059). These findings suggest that exosomal miR‐493, by downregulating MAD2L1, contributes to PTX resistance and poorer prognosis [79]. The prognostic value of MAD2L1 expression, modulated by miR‐493, suggests its potential as a biomarker for identifying GC patients at risk of treatment failure. However, its role in guiding personalised therapeutic strategies, such as alternative chemotherapeutic agents or miR‐493‐targeted interventions, requires further investigation.

As reported by Jing et al., elevated exosomal miR‐769‐5p levels are strongly associated with CDDP resistance in GC, as evidenced by significantly higher expression in CDDP‐resistant patients' serum exosomes (*n* = 19) compared to CDDP‐sensitive patients (*n* = 41; *p* < 0.05). In clinical samples (*n* = 75 pairs), miR‐769‐5p expression was markedly elevated in GC tissues compared to para‐cancerous tissues (*p* < 0.0001), correlating with advanced TNM stage and poor prognosis. These findings, supported by TCGA data (*n* = 346) showing higher miR‐769‐5p in GC tissues (*p* < 0.0001), suggest that exosomal miR‐769‐5p contributes to CDDP resistance and disease progression, likely by targeting CASP9 and promoting p53 ubiquitination degradation [100]. The prognostic value of miR‐769‐5p positions it as a potential non‐invasive biomarker for identifying GC patients at risk of treatment failure and poor survival, potentially guiding personalised therapeutic strategies, such as alternative chemotherapies or miR‐769‐5p‐targeted interventions.

Exosomal miR‐500a‐3p levels in plasma are strongly associated with CDDP resistance in stage III GC patients, as evidenced by significantly higher expression in both plasma exosomes and GC tissues of CDDP‐resistant patients compared to CDDP‐sensitive patients (*p* < 0.05) [82]. It showed that high exosomal miR‐500a‐3p expression negatively correlated with prognosis, as demonstrated by Kaplan–Meier analysis showing poorer survival outcomes in patients with elevated levels. Receiver Operating Characteristic (ROC) curve analysis revealed that plasma exosomal miR‐500a‐3p levels discriminated between resistant and sensitive groups with high accuracy (AUC = 0.843) [82]. These findings suggest that miR‐500a‐3p, by negatively regulating FBXW7, promotes CDDP resistance and stemness, contributing to adverse prognosis (Table 4). Exosomal miR‐500a‐3p has demonstrated prognostic potential as a non‐invasive biomarker for identifying GC patients at risk of treatment failure and poor survival, though its clinical application in guiding personalised therapies remains to be validated.

According to Qu et al., decreased levels of exosomal lncRNA DACT3‐AS1 are strongly associated with poor prognosis and oxaliplatin resistance in GC, as evidenced by significantly reduced expression in GC tumour tissues compared to paracancerous tissues in 93 paired samples (*p* < 0.05) and in the TCGA database (*p* < 0.05) [27]. Low DACT3‐AS1 expression correlated with adverse clinical features, including larger tumour size, advanced stage, poorly differentiated pathology and lymph node metastasis, and was linked to poor prognosis in Kaplan–Meier analysis (GSE15459: HR = 0.64 (0.42–0.99), *p* = 0.046; GSE22377: HR = 0.24 (0.1–0.55), *p* = 0.00027). ROC analysis demonstrated that DACT3‐AS1 expression discriminated between tumour and non‐tumour tissues with moderate accuracy (AUC = 0.6940, *p* < 0.001) [27]. These findings suggest that downregulation of DACT3‐AS1, possibly from CAFs, promotes malignant transformation and ferroptosis‐mediated oxaliplatin resistance, leading to worse survival outcomes. DACT3‐AS1 serves as a potential biomarker for identifying gastric cancer patients at risk of poor prognosis and treatment failure (Table 4).

miR‐374a‐5p levels increasing in serum, has been reported by Ji et al. are associated with poor prognosis and oxaliplatin resistance in GC, as evidenced by significantly higher expression in GC patients (*n* = 59) compared to gastritis patients and healthy controls (*n* = 34; *p* < 0.05) [84]. Serum miR‐374a‐5p was approximately 5‐fold higher in patients with pre‐cancerous lesions compared to controls and was significantly reduced post‐operatively but elevated in relapsed patients (*p* < 0.05). ROC analysis demonstrated high diagnostic accuracy (AUC = 0.919, 95% CI: 0.866–0.972) [84]. High miR‐374a‐5p levels, particularly in larger tumours (> 5 cm), suggest its role in chemoresistance and disease progression, positioning it as a potential non‐invasive biomarker for identifying GC patients at risk of poor prognosis and treatment failure, guiding targeted therapeutic strategies.

ALOX15 promotes ferroptosis via Lipid‐ROS, improving OS in GC patients in a dose‐dependent manner. miR‐522, secreted by CAFs via USP7/hnRNPA1‐enhanced exosomes, inhibits ALOX15, leading to reduced ferroptosis and poorer OS in high miR‐522 groups (*p* < 0.05). Chemotherapy agents, such as CDDP and PTX, further increase miR‐522 secretion by activating USP7/hnRNPA1, thereby exacerbating resistance. Silencing USP7/hnRNPA1/miR‐522 in CAFs enhances ferroptosis, slows tumour growth and extends survival in mice. Targeting this axis may improve ALOX15‐driven ferroptosis and chemotherapy response (*p* < 0.05) [101].

In GC patients, Zang et al. reported that higher expression levels of circ50547 were significantly correlated with clinicopathological features indicative of poor prognosis, including lymphatic metastasis (*p* = 0.0193), invasion depth (*p* = 0.0123) and advanced TNM stage (*p* = 0.0047), highlighting its potential as a prognostic biomarker in GC [112].

Analysing TCGA survival data for GC patients undergoing chemotherapy, Kong et al. found that higher expression of miR‐1911–5p was significantly correlated with poorer prognosis (*p* = 0.017), suggesting its potential as a prognostic biomarker for chemotherapy outcome [103]. Moreover, Analysing TCGA database data for GC, Dong et al. revealed that higher expression levels of miR‐9‐3p were negatively associated with patient disease‐specific survival (DSS), highlighting its potential as a prognostic biomarker in GC [102] (Table 4).

#### 3.3.5 ESCC

Referring to Table 5, serum exosomal ncRNAs in ESCC with focusing on their predictive value for radiotherapy and chemotherapy resistance have been studied and significant correlations with poor survival have been shown.

In the ESCC patients, elevated serum levels of miR‐143‐3p (> 1.5‐fold) are strongly associated with radiotherapy resistance, as evidenced by shorter PFS (*p* = 0.008) and OS (*p* = 0.024) in the resistant group. The combination of miR‐143‐3p and miR‐223‐3p enhances prognostic accuracy (AUC: 0.703 for OS, 0.751 for PFS), suggesting that miR‐143‐3p could serve as a non‐invasive biomarker for identifying patients at risk of early recurrence or poor survival. The independent prognostic value of miR‐143‐3p in multivariate analysis underscores its potential clinical relevance and warrants further investigation into its role in guiding personalised treatment strategies, such as intensified radiotherapy or exosome‐targeted therapies [92].

Based on the study of Tong et al. elevated plasma exosomal lncRNA POU3F3 levels are strongly associated with CDDP resistance in ESCC, as evidenced by significantly higher expression in patients without a complete response (CR) to CDDP‐based chemoradiotherapy compared to those achieving CR (*p* < 0.001). Among 138 ESCC patients, only 25.3% (35/138) achieved CR and pre‐treatment plasma exosomal lncRNA POU3F3 was significantly lower in CR patients (*p* < 0.001) [94].

Additionally, higher pre‐treatment lncRNA POU3F3 levels correlated with poorer OS (*p* < 0.001) in 78 postoperative recurrent ESCC patients receiving CDDP ‐based chemotherapy. Multivariate analysis confirmed lncRNA POU3F3 as an independent predictor of poor OS, underscoring its prognostic value [94]. The combination of lncRNA POU3F3 expression with clinical outcomes suggests it could serve as a non‐invasive biomarker for identifying patients at risk of CDDP resistance and poor survival, potentially guiding personalised treatment strategies, such as alternative chemotherapy regimens or therapies targeting exosome‐mediated pathways.

In a cohort of 174 surgically treated ESCC patients, Zhou et al. observed that a high stromal presence of FAP‐positive CAFs (lncRNA AFAP1‐AS1) was significantly associated with shorter PFS (*p* < 0.001) and OS (*p* < 0.001), indicating FAP‐positive CAFs as a strong negative prognostic biomarker [106].

## 4 Discussion

This systematic review (76 studies) synthesises the role of exosomal ncRNAs in drug resistance across GI malignancies (CRC, GC, HCC, PDAC, ESCC). Our synthesis reveals ncRNAs converge on survival pathways (e.g., PI3K/AKT, Wnt/β‐catenin), regulate cell fate (apoptosis, autophagy, ferroptosis) and mediate TME‐driven resistance via CAF/TAM exosomes. These findings posit drug resistance as an ecosystemic, not solely cell‐intrinsic, phenomenon. On the basis of reviewed articles some ncRNAs including miR‐208b, miR‐24‐3p, miR‐92a‐3p, miR‐196b‐5p, miR‐181d‐5p, FAL1, CACClnc, CCAL, circ_0067557 and circ_0004771 that correlate with poor prognosis in CRC patients. For example, Ning et al. reported that miR‐208b suppresses PDCD4 resulting in decreasing PFS [39]. Previosusly, PDCD4 was suggested to act as a tumour suppressor through apoptosis induction, inhibition of proteogenesis and suppressing AP‐1 that is responsible for metastasis, invasion and cell proliferation [113]. The potential of exosomal ncRNAs as prognostic and predictive biomarkers is a recurring theme. While individual molecules like miR‐208b correlate with poor prognosis in CRC [39], the field is advancing toward multi‐marker panels with enhanced clinical utility. A landmark study by Xu et al. developed a 14‐miRNA exosomal signature that robustly predicted patient response to anti‐EGFR therapy in metastatic CRC [97]. This panel's predictive power was linked to the modulation of the TP53‐mediated apoptosis cascade, demonstrating a clear mechanistic underpinning for its clinical performance and highlighting the promise of liquid biopsies for personalising treatment strategies.

miR‐3173‐5p, miR‐155 and circZNF91 association with reduced OS/DFS in PDAC has been reported [65, 68, 70]. In the HCC patients circCCAR1, circUPF2 and circDCAF8 are linked to reduced OS and DFS [75, 76, 77], while circ‐G004213 enhances CDDPCCDDP sensitivity, improving survival [78].

In ESCC patients it is showed that miR‐143‐3p correlates with radiotherapy resistance and reduced PFS/OS [92] and lncRNA POU3F3 is associated with CDDPCCDDP resistance and reduced OS [94]. Moreover, miR‐769‐5p [100], miR‐500a‐3p [82] and miR‐374a‐5p [84] are related to CDDP/oxaliplatin resistance and reduced OS/PFS; also, downregulated lncRNA DACT3‐AS1 correlates with poor prognosis and oxaliplatin resistance [27].

The role in drug resistance of exosome driven ncRNAs in each GI cancer could act through different axis. For instance, in CRC patients, it is shown that miR‐24‐3p, miR‐625‐3p and miR‐92a‐3p promote resistance via CDX2/HEPH, CELF2/WWOX and Wnt/β‐catenin pathways. This suggests that CDX2 modulates the expression of the HEPH in the epithelial cells of the colon as a transcription factor [114]. The induction of HEPH modulated by CDX2 and suppression of the CDX2/HEPH axis results in chemoresistance in cancer cells of the colon [114]. CELF2 targets the WWOX and acts as a TSG, and suppression of CELF2 leads to cell proliferation and drug resistance [109]. Further diversifying these resistance mechanisms in CRC, Yan et al. demonstrated that chemoresistance can also be modulated through the disruption of calcium signalling pathways [98]. They found that a decrease in exosomal miR‐224‐5p leads to the upregulation of its target, S100A4, thereby enhancing malignant properties and resistance to 5‐FU.

Furthermore, our study reveals that exosomal ncRNAs modulate resistance through mechanisms extending beyond metabolic and survival signalling, such as the regulation of DNA damage repair. A prime example is the transfer of exosomal lncRNA AFAP1‐AS1 from a distinct subpopulation of FAP‐positive cancer‐associated fibroblasts, which was shown to enhance radioresistance in oesophageal squamous cell carcinoma by promoting more efficient DNA repair mechanisms [106]. Overall, these findings show that drug resistance is an ecosystemic phenomenon, cooperative by a complex interplay of cell‐intrinsic and TME‐derived signals.

Several studies showed that many signalling pathways are implicated to varying degrees in the development of drug resistance or poor prognosis of GI cancers. The most important key signalling pathways and proteins include Wnt/β‐catenin, PI3K/AKT, NF2/Hippo, STAT3, mTOR, FoxO/AMPK, JAK2, HIF‐1α, autophagy, ferroptosis and p53.

The Wnt/β‐catenin axis has a crucial role in physiological development; on the other hand, this signalling pathway is linked to the development of CRC. Aberrant activation of Wnt/β‐catenin is the result of APC gene mutations implicated in tumorigenesis and cancer progression. This activation elevates cell proliferation, reduces apoptosis and increases metastasis [115]. In our study, it showed that miR‐24‐3p, miR‐625‐3p and miR‐92a‐3p induce CRC drug resistance and poor prognosis through activating this pathway.

PI3K/AKT regulates survival/apoptosis in CRC, PDAC and HCC. For instance, it was demonstrated that the PI3K/AKT pathway is crucial in CRC progression by regulating EMT. This axis leads to the induction of mTOR, Rac1 and RhoA in cooperation with proteins involved in the extracellular matrix and cytoskeleton, resulting in cell invasion properties [116]. Moreover, it is suggested by some studies that one of the results of PI3K/AKT axis activation is drug resistance induction due to promoting ABC transporters through drug efflux enhancement in CRC patients [117, 118]. This signalling pathway could be activated through upregulation of miR‐184, miR‐100, miR‐92a and miR‐16 and downregulation of miR‐30e, miR‐144‐5p and let‐7i as shown in our investigation in CRC patients, resulting in drug resistance. The cross‐cancer relevance of these core signalling pathways is powerfully exemplified by recent findings in HCC. A study by Cao et al. revealed a sophisticated resistance mechanism driven by a single exosomal lncRNA, lnc‐FAM72D‐3. This molecule was shown to confer Lenvatinib resistance by simultaneously co‐activating both the Wnt/β‐catenin pathway and the PI3K/AKT/mTOR signalling cascade [105]. This finding is particularly significant as it illustrates how a single exosomal signal can hijack multiple fundamental survival and proliferation pathways, creating a robust and multifaceted resistance phenotype. It declares that these signalling axes often function as an interconnected network, and targeting them may have broad applicability across different GI malignancies characterised by exosome‐mediated therapeutic failure.

Beyond these well‐established oncogenic pathways, our study also uncovers the dysregulation of the Hippo signalling pathway as an emerging axis of chemoresistance. Notably, a study by Zhao et al. in CRC identified that exosomal miR‐223‐3p confers 5‐FU resistance by directly targeting the core Hippo component NF2 [99]. The disruption of the Hippo pathway as a critical regulator of organ size, tissue homeostasis and cell proliferation highlights another fundamental biological process subverted by exosome‐mediated intercellular communication to promote therapeutic failure.

The STAT3 pathway, another chemoresistance mechanism in CRC, can be induced by ncRNAs. Its activation via Abl/Src, Src/JAK and JAK/JAK factors leads to STAT3 promoter binding, ultimately resulting in immune suppression and metastasis [119]. As reported in this study, miR‐196b‐5p could enhance drug resistance in CRC patients. A study suggested that miR‐196b‐5p could elevate the IL‐6 levels through inhibiting FAS ligand, leading to interfere with the suppression of NF‐κB and to elevate IL‐6 transcription, resulting in higher activation of STAT3 and cell proliferation [120].

Our analysis identified mTOR as a key modulator of drug resistance in HCC and CRC, where its activation via the PI3K/AKT pathway promotes EMT and progression. Mechanistically, PI3K phosphorylates PIP2 to PIP3, recruiting AKT/PDK1 to activate mTORC1, which in turn increases protein synthesis and activates BAD/NF‐κB pathways, driving cell survival and metastasis in CRC [121]. Activation of mTOR in the ESCC is also referred to hyperactivity of the pentose phosphate shunt, which leads to higher levels of NADPH that suppress the ROS and interferes with the AMPK suppression by ROS, resulting in elevating mTOR and cell proliferation [59].

Exosomal ncRNAs modulate the FoxO/AMPK pathway in CRC patients. According to Beretta et al., FoxO proteins are transcription factors regulating cell cycle, apoptosis and drug resistance genes. Their activity is regulated by post‐translational modifications (e.g., phosphorylation, ubiquitination), and their expression levels are affected by ncRNAs such as miRNAs [122]. The cancer induction effect of FoxO showed that performs through different ways such as drug resistance, apoptosis, cell‐cycle arrest and anti‐oxidant response modulations. For instance, FoxO induce drug resistance via modulating of ABCB1, ABCC2, JNK, PI3K/AKT, ERK1/2 and TRX1 [122].

HIF‐1α has been suggested as a predictive factor of drug resistance in PDAC patients. HIF‐1α has a crucial role in tumour biology as a transcription factor that modulates the expression of numerous genes, and these genes are implicated in the adaptation and survival of cancer cells during hypoxia [123]. Hypoxia‐induced HIF‐1α promotes tumour growth (by modulating pH homeostasis, apoptosis, angiogenesis, survival) and metastasis (by inducing proteins like Snail, MMPs, ZEB1, LOX). It also confers radio/drug resistance by inhibiting DNA damage/apoptosis and activating autophagy, tumour metabolism and drug efflux [124]. For example, in PDAC patients it has been demonstrated that HIF‐1α mediates the upregulation of MDR1 leading to TME immunosuppression and chemotherapy resistance in PDAC patients accompanied with poor prognosis [125, 126].

JAK2, a tyrosine kinase receptor modulating cell proliferation, differentiation and survival, reportedly enhances CDDP resistance in ESCC, where JAK2/STAT3 pathway inhibition suppresses cell growth and cancer‐associated inflammation [127]. Pandey et al. showed that there are multiple ways to activate the JAK2 pathway; the most possible ways are heterodimerisation with JAK1/TYK2 and JAK2‐independent activation, resulting in activation of the RAS–ERK pathway leading to cell proliferation and cytokine production [128]. The heterodimerisation with JAK1/TYK2 way claimed that it could induce drug resistance by affecting BAD/BCL‐XL proteins [128].

Our analysis indicates ferroptosis induces drug sensitivity in GC and PDAC. This iron‐dependent cell death, driven by lipid peroxide accumulation, modulates drug resistance via iron/lipid metabolism. Inducing ferroptosis (e.g., via GPX4 inhibitors, ROS, iron) enhances treatment sensitivity, an effect also seen with immunotherapy, which increases lipid peroxidation [129]. Various mechanisms implicated in drug resistance of tumours, of which oxidative homeostasis imbalance is a crucial factor. By suppressing ROS production, tolerance of tumour cells to oxidative stress will increase and develop acquired drug resistance. The dynamic balance between production of ROS and elimination is determining: ROS and lipid peroxidation elevation induce ferroptosis and restrict tumour proliferation, while their reduction promotes tumour cell survival and resistance to antitumour drugs [129]. Zhang et al. introduced three pathways for inducing ferroptosis to reverse drug resistance. The GPX4 pathway involves suppressing GPX4 by inhibiting glutathione biosynthesis and cysteine uptake. The iron metabolism pathway increases the cellular Labile Iron Pool (LIP) via DHA and DMT1/LCN2 downregulation. Finally, the lipid metabolism pathway targets ACSL4 through ARF6 downregulation and LOX inhibition, all inducing ferroptosis and chemosensitivity [130]. Our analysis identifies ferroptosis as a critical regulatory node for modulating drug sensitivity, a concept strongly reinforced by recent studies focusing on the TME in GC. Compelling evidence from Dong et al. demonstrates that TANs release exosomes enriched with miR‐9‐3p [102]. This miRNA directly targets ACSL4, a key mediator of lipid peroxidation, thereby inhibiting ferroptosis and conferring resistance to oxaliplatin. Complementing this finding, Kong et al. revealed a similar mechanism involving M2‐polarised TAMs, which secrete exosomal miR‐1911–5p [103]. This ncRNA shields GC cells from cisplatin‐induced ferroptotic death by suppressing the MYB/AKR1B10/ACC signalling cascade. In addition to the TME‐driven suppression of ferroptosis, drug resistance in GC is also promoted by intricate circular RNA‐driven ceRNA networks. For instance, Zang et al. recently identified that exosomal circ50547 fosters oxaliplatin resistance by acting as a sponge for miR‐217, consequently leading to the derepression of its target, HNF1B [112]. These studies show TME immune cells employ functionally diverse exosomal ncRNAs to remodel cancer cell metabolism (via metabolic reprogramming and gene regulation), establishing ferroptosis suppression as a convergent, clinically significant chemoresistance mechanism in GI cancers.

Autophagy, a self‐degradative process, shows a dual role in cancer treatment. As discussed herein for CRC and GC, it can promote drug resistance and survival by recycling dysfunctional components. However, autophagy may also be triggered by these therapies, potentially resulting in cell death, thus highlighting its dual role in cancer treatment [131]. The mechanism of autophagy‐mediated drug resistance involves: **Initiation** by the ULK1/2 complex (regulated by mTOR, AMPK, MAPK); **Nucleation** by the Beclin1/VPS34 complex (inhibited by Bcl‐2/Bcl‐XL); and **Elongation**. This final step uses the ATG12/ATG5/ATG16 complex and ligases (ATG7, 10, 3) for the phosphatidylethanolamine (PE) lipidation of LC3‐I to LC3‐II. This process promotes MDR by forming autolysosomes that degrade drug‐containing cargo [131]. In each step of this process, various ncRNAs are implicated [131], which show the potential effect of exosomal ncRNAs in the drug resistance problem.

A notable finding from this analysis is the identification of exosomal ncRNAs (Figure 2) with multi‐functional biomarker potential, possessing both prognostic (predicting survival) and predictive (predicting therapy response) capabilities. This dual capacity represents a critical step toward personalised oncology. However, this potential is tempered by significant translational caveats. Robust validation is currently impeded by profound methodological and clinical heterogeneity (e.g., inconsistent isolation techniques and non‐standardised resistance definitions). Furthermore, the evidence base is overwhelmingly derived from East Asian cohorts, raising critical questions about generalisability. Most critically, a substantial translational gap persists, as none of the identified biomarkers have transcended preclinical validation and achieved clinical utility.

## 5 Study Limitations

There are several limitations to this systematic review that should be acknowledged. First, the review was based solely on studies published in indexed journals, which may introduce both publication bias and time‐lag bias. In particular, studies reporting negative or inconclusive results may be delayed or underrepresented in the literature compared to those with more favourable outcomes [132].

Additionally, it is important to note that all patients included in the reviewed studies were from China, which may limit the generalisability of these findings to other populations and ethnic groups.

Second, the literature search was restricted to English‐language publications, introducing a potential language bias by excluding relevant studies published in other languages.

Third, studies involving animal models, cell lines, or purely in vitro experiments without validation in human samples were excluded. While this was done to maintain clinical relevance, it may have resulted in the omission of valuable mechanistic data.

Moreover, the possibility of non‐differential misclassification exists, as some individuals classified within control groups in the included studies might develop cancer later in life, potentially influencing study outcomes.

Finally, most of the data included were based on unadjusted analyses. Future studies incorporating adjusted estimates that account for potential confounders, such as age, tobacco use, alcohol consumption and environmental exposures, would likely provide a more precise and reliable assessment of the associations reported.

## 6 Conclusions and Future Perspectives

Investigation of exosomal ncRNAs, including miRNAs, lncRNAs and circRNAs, has revealed their potential key role in drug resistance and poor prognosis in GI cancers. These ncRNAs, located within exosomes, act as mediators of intercellular communication, regulating key signalling pathways such as Wnt/β‐catenin, PI3K/AKT, STAT3, mTOR, autophagy and ferroptosis, leading to chemotherapy resistance and poor clinical outcomes. In colorectal, pancreatic, liver, gastric and oesophageal cancers, exosomal ncRNAs enhance resistance to chemotherapeutic drugs and reduce patient survival. Autophagy serves as a survival mechanism against chemotherapy, while ferroptosis can improve therapeutic sensitivity and ncRNAs have potential as non‐invasive biomarkers to predict treatment failure and personalise therapies.

The dual role of exosomal ncRNAs, particularly in modulating autophagy and ferroptosis, highlights their complex influence on treatment response. These findings highlight the potential of exosomal ncRNAs as non‐invasive biomarkers for predicting treatment failure and stratifying patients for personalised therapeutic strategies. However, the lack of studies on exosomal lncRNAs in HCC and the scarcity of data on ESCC highlight significant research gaps in variations of drug/radiation resistance, emphasising a critical need for future investigations.

Future investigations should focus on elucidating the mechanistic interplay of ncRNAs across diverse GI cancers and validating their clinical utility in large, multi‐ethnic cohorts. Targeting exosomal ncRNAs or their downstream pathways, such as through miRNA inhibitors or ferroptosis inducers, holds promise for overcoming drug resistance and improving survival outcomes in GI cancer patients. Furthermore, future studies should also focus on integrating these biological findings with computational models and leveraging public datasets (e.g., TCGA, GEO) to develop robust predictive algorithms for drug resistance.

## Author Contributions

M.S., Z.S., F.B. and N.F. contributed to the conceptualisation of the study. M.S., Z.S., F.B., S.A. and N.F. collected references and performed the initial literature search. M.S., Z.S., F.B., S.A., N.F. and S.N. wrote the msanuscript. M.S., Z.S. and F.B. prepared the tables, and S.A. and N.F. prepared the figures. N.F., M.S., Z.S., F.B., A.S. and S.N. revised the article. All authors read and approved the final manuscript.

## Funding

The authors have nothing to report.

## Consent

This systematic review did not require ethics approval, as no animal or human subjects were used in this study.

## Conflicts of Interest

The authors declare no conflicts of interest.

## Supporting information


**Data S1:** (A) Script for searching in the PubMed (*n* = 220). (B) Script for searching in the Scopus (*n* = 180). (C) Script for searching in the Web of Science (*n* = 177).

**Table S1:** Results from the Critical Appraisal Skills Programme quality assessment checklist for case control studies.

**Table S2:** Results from the Critical Appraisal Skills Programme quality assessment checklist for cohort studies.

## Data Availability

All data generated or analysed during this study are included in this published article [and its File S1].
