# Supplementary material for: Exosomal Non‐Coding RNAs in Gastrointestinal Cancer Drug Resistance: A Systematic Review of Emerging Mechanisms and Clinical Implications
Source: J Cell Mol Med. 2026 May 8;30(9):e71137. doi: 10.1111/jcmm.71137 (PMC13156249; doi:10.1111/jcmm.71137)
Supplement: Supplementary file 1 — Data S1: (A) Script for searching in the PubMed (n = 220). (B) Script for searching in the Scopus (n = 180). (C) Script for searching in the Web of Science (n = 177). [file JCMM-30-e71137-s002.docx]

**A. Script for searching in the PubMed (n=220)**

**(("gastrointestinal neoplasms"[MeSH Terms] OR "colorectal neoplasms"[MeSH Terms] OR "pancreatic neoplasms"[MeSH Terms] OR "stomach neoplasms"[MeSH Terms] OR "liver neoplasms"[MeSH Terms] OR "esophageal neoplasms"[MeSH Terms] OR "gastrointestinal cancer*"[Title/Abstract] OR "colorectal cancer*"[Title/Abstract] OR "pancreatic cancer*"[Title/Abstract] OR "gastric cancer*"[Title/Abstract] OR "stomach cancer*"[Title/Abstract] OR "liver cancer*"[Title/Abstract] OR "carcinoma, hepatocellular"[MeSH Terms] OR "esophageal cancer*"[Title/Abstract]) AND ("exosomes"[MeSH Terms] OR "exosomal"[Title/Abstract] OR "exosome-derived"[Title/Abstract] OR "exosome"[Title/Abstract]) AND ("rna, untranslated"[MeSH Terms] OR "rna, long noncoding"[MeSH Terms] OR "rna, circular"[MeSH Terms] OR "ncrnas"[Title/Abstract] OR "mirnas"[Title/Abstract] OR "lncrnas"[Title/Abstract] OR "circrnas"[Title/Abstract] OR "pirnas"[Title/Abstract]) AND ("drug resistance, neoplasm"[MeSH Terms] OR "drug resistance"[Title/Abstract] OR "chemoresistance"[Title/Abstract] OR "therapy resistance"[Title/Abstract]) AND 1900/01/01:2025/10/18[Date - Publication])**

**B. Script for searching in the Scopus (n=180)**

**TITLE-ABS-KEY (( "gastrointestinal neoplasm*" OR "gastrointestinal cancer*" OR "colorectal neoplasm*" OR "colorectal cancer*" OR "pancreatic neoplasm*" OR "pancreatic cancer*" OR "gastric neoplasm*" OR "gastric cancer*" OR "stomach cancer*" OR "liver neoplasm*" OR "liver cancer*" OR "hepatocellular carcinoma" OR "esophageal neoplasm*" OR "esophageal cancer*" ) AND ( "exosome*" OR "exosomal" OR "exosome-derived" ) AND ( "noncoding rna*" OR "ncrna*" OR "microrna*" OR "mirna*" OR "long noncoding rna*" OR "lncrna*" OR "circular rna*" OR "circrna*" OR "piwi-interacting rna*" OR "pirna*" ) AND ( "drug resistance" OR "chemoresistance" OR "therapy resistance" OR "treatment resistance" ) AND PUBYEAR > 1899 AND PUBYEAR < 2026**

**C. Script for searching in the Web of Science (n=177)**

**TS=(("gastrointestinal neoplasm*" OR "gastrointestinal cancer*" OR "colorectal neoplasm*" OR "colorectal cancer*" OR "pancreatic neoplasm*" OR "pancreatic cancer*" OR "gastric neoplasm*" OR "gastric cancer*" OR "stomach cancer*" OR "liver neoplasm*" OR "liver cancer*" OR "hepatocellular carcinoma" OR "esophageal neoplasm*" OR "esophageal cancer*") AND ("exosome*" OR "exosomal" OR "exosome-derived") AND ("noncoding rna*" OR "ncrna*" OR "microrna*" OR "mirna*" OR "long noncoding rna*" OR "lncrna*" OR "circular rna*" OR "circrna*" OR "piwi-interacting rna*" OR "pirna*") AND ("drug resistance" OR "chemoresistance" OR "therapy resistance" OR "treatment resistance") AND PY=(1900-2025)**
