# Supplementary material for: Exosomal Non‐Coding RNAs in Gastrointestinal Cancer Drug Resistance: A Systematic Review of Emerging Mechanisms and Clinical Implications
Source: J Cell Mol Med. 2026 May 8;30(9):e71137. doi: 10.1111/jcmm.71137 (PMC13156249; doi:10.1111/jcmm.71137)
Supplement: Supplementary file 2 — Table S1: Results from the Critical Appraisal Skills Programme quality assessment checklist for case control studies. [file JCMM-30-e71137-s001.docx]

Supplementary Table 1. Results from the Critical Appraisal Skills Programme quality assessment checklist for case control studies.

| **Study** | **Did the study address a clearly focused issue?** | **Did the authors use an appropriate method to answer their question?** | **Were the cases recruited in an acceptable way?** | **Were the controls selected in an acceptable way?** | **Was the exposure accurately measured to minimize bias?** | **Have the authors taken account of the potential confounding factors in the design and/or in their analysis?** | **Do you believe the results?** | **Can the results be applied to the local population?** | **Do the results of this study fit with other available evidence?** | **How valuable is the research?** |
| --- | --- | --- | --- | --- | --- | --- | --- | --- | --- | --- |
| **Zhang, Y., et al. (2022)** | Yes | Yes | Yes | Yes | Yes | No | Yes | Yes | Yes | Valuable |
| **Hui, B., et al. (2022)** | Yes | Yes | Yes | Yes | Yes | Yes | Yes | Yes | Yes | Valuable |
| **Zhao, K., et al. (2021)** | Yes | Yes | Yes | Yes | Yes | Yes | Yes | Yes | Yes | Valuable |
| **Xu, Y., et al. (2021)** | Yes | Yes | Yes | Yes | Yes | Yes | Yes | Yes | Yes | Valuable |
| **Wang, X., et al. (2020)** | Yes | Yes | Yes | Yes | Yes | Can’t Tell | Yes | Yes | Yes | Valuable |
| **Hon, K. W., et al. (2019)** | Yes | Yes | Yes | Yes | Yes | Yes | Yes | Yes | Yes | Valuable |
| **Xiao-Xue Qiao, et al. (2023)** | Yes | Yes | Yes | Yes | Yes | Yes | Yes | Yes | Yes | Valuable |
| **Zihao Pan, et al. (2022)** | Yes | Yes | Yes | Yes | Yes | Yes | Yes | Yes | Yes | Valuable |
| **Zhan Qu, et al. (2022)** | Yes | Yes | Yes | Yes | Yes | Can’t Tell | Yes | Yes | Yes | Valuable |
| **Chen Li, X. L. (2022)** | Yes | Yes | Yes | Yes | Yes | No | Yes | Yes | Yes | Valuable |
| **Xijuan Chen, et al. (2021)** | Yes | Yes | Yes | Yes | Yes | No | Yes | Yes | Yes | Valuable |
| **Xijuan Chen, et al. (2021)** | Yes | Yes | Yes | Yes | Yes | No | Yes | Yes | Yes | Valuable |
| **Jiayi Han, et al. (2020)** | Yes | Yes | Yes | Yes | Yes | Yes | Yes | Yes | Yes | Valuable |
| **Guoying Jin, et al. (2019)** | Yes | Yes | Yes | Yes | Yes | Yes | Yes | Yes | Yes | Valuable |
| **Ying-nan Yang, et al. (2018)** | Yes | Yes | Yes | Yes | Yes | Yes | Yes | Yes | Yes | Valuable |
| **Feng-Lin Dong, et al. (2024)** | Yes | Yes | Yes | Yes | Yes | Yes | Yes | Yes | Yes | Valuable |
| **Biao Tang, et al. (2023)** | Yes | Yes | Yes | Yes | Yes | Yes | Yes | Yes | Yes | Valuable |
| **Lin Xin, et al. (2021)** | Yes | Yes | Yes | Yes | Yes | Yes | Yes | Yes | Yes | Valuable |
| **Huijie Gao, et al. (2020)** | Yes | Yes | Yes | Yes | Yes | Yes | Yes | Yes | Yes | Valuable |
| **Runbi Ji, et al. (2019)** | Yes | Yes | Yes | Yes | Yes | Yes | Yes | Yes | Yes | Valuable |
