# Supplementary material for: Exosomal Non‐Coding RNAs in Gastrointestinal Cancer Drug Resistance: A Systematic Review of Emerging Mechanisms and Clinical Implications
Source: J Cell Mol Med. 2026 May 8;30(9):e71137. doi: 10.1111/jcmm.71137 (PMC13156249; doi:10.1111/jcmm.71137)
Supplement: Supplementary file 3 — Table S2: Results from the Critical Appraisal Skills Programme quality assessment checklist for cohort studies. [file JCMM-30-e71137-s003.docx]

Supplementary Table 2. Results from the Critical Appraisal Skills Programme quality assessment checklist for cohort studies.

| Study | Did the study address a clearly focused issue? | Was the cohort recruited in  an acceptable way? | Was the exposure accurately  measured to minimise bias? | Was the outcome accurately  measured to minimise bias? | Have the authors identified  all important confounding  factors? | Have they taken account of  the confounding factors in the  design and/or analysis? | Was the follow up of  subjects complete enough? | Was the follow up of  subjects long enough? | Do you believe the results? | Can the results be applied to  the local population? | Do the results of this study fit  with other available  evidence? | What are the implications of  this study for practice? | How valuable is the research? |
| --- | --- | --- | --- | --- | --- | --- | --- | --- | --- | --- | --- | --- | --- |
| Zhu, S., et al. (2024) | Yes | Yes | Yes | Yes | Yes | Yes | Yes | Yes | Yes | Yes | Yes | Yes | Valuable |
| Yang, C., et al. (2024) | Yes | Yes | Yes | Yes | Can’t Tell | No | No | No | Yes | Yes | Yes | Yes | Valuable |
| Zhang, X., et al. (2023) | Yes | Yes | Yes | Yes | Yes | Yes | Yes | Yes | Yes | Yes | Yes | Yes | Valuable |
| Huang, C., et al. (2023) | Yes | Yes | Yes | Yes | No | No | Yes | Yes | Yes | Yes | Yes | Yes | Valuable |
| Deng, J., et al. (2023) | Yes | Yes | Yes | Yes | Yes | Can’t Tell | No | No | Yes | Yes | Yes | Yes | Valuable |
| Zhang, H. W., et al. (2021) | Yes | Yes | Yes | Yes | Can’t Tell | Yes | Can’t Tell | Can’t Tell | Yes | Yes | Yes | Yes | Valuable |
| Ning, T., et al. (2021) | Yes | Yes | Yes | Yes | Can’t Tell | Yes | Can’t Tell | Can’t Tell | Yes | Yes | Yes | Yes | Valuable |
| Deng, X., et al. (2020) | Yes | Yes | Yes | Yes | Can’t Tell | Yes | Can’t Tell | Can’t Tell | Yes | Yes | Yes | Yes | Valuable |
| Hu, J. L., et al. (2019) | Yes | Yes | Yes | Yes | No | No | Can’t Tell | Can’t Tell | Yes | Yes | Yes | Can’t Tell | Valuable |
| Ren, J., et al. (2018) | Yes | Yes | Yes | Yes | Can’t Tell | Can’t Tell | Can’t Tell | Can’t Tell | Yes | Yes | Yes | Can’t Tell | Valuable |
| Ren, D., et al. (2017) | Yes | Yes | Yes | Yes | No | Can’t Tell | Yes | Yes | Yes | Yes | Can’t Tell | Yes | Valuable |
| Jun-Hong Hu, et al. (2024) | Yes | Yes | Yes | Yes | Can’t Tell | Can’t Tell | Can’t Tell | Can’t Tell | Yes | Yes | Yes | Can’t Tell | Valuable |
| Wenjing Gong, et al. (2023) | Yes | Yes | Yes | Yes | No | No | Yes | Yes | Yes | Yes | Yes | Yes | Valuable |
| Ying Zhang, et al. (2022) | Yes | Yes | Yes | Yes | No | No | No | No | Yes | Yes | Yes | Yes | Valuable |
| Shengli Pan, et al. (2022) | Yes | Yes | Yes | Yes | Yes | Yes | Yes | Yes | Yes | Yes | Yes | Yes | Valuable |
| Takahiro Yagi, et al. (2019) | Yes | Yes | Yes | Yes | Yes | Yes | No | No | Yes | Yes | Yes | Yes | Valuable |
| Ran Qi, et al. (2023) | Yes | Yes | Yes | Yes | Yes | Yes | Yes | Yes | Yes | Yes | Yes | Yes | Valuable |
| Zhu Zeng, et al. (2021) | Yes | Yes | Yes | Yes | Yes | Yes | Yes | Yes | Yes | Yes | Yes | Yes | Valuable |
| Yuan Chi, et al. (2021) | Yes | Yes | Yes | Yes | Yes | Yes | Can’t Tell | Can’t Tell | Yes | Yes | Yes | Yes | Valuable |
| Yan Xu, et al. (2020) | Yes | Yes | Yes | Yes | Yes | Yes | Can’t Tell | Can’t Tell | Yes | Yes | Can’t Tell | Yes | Valuable |
| Ming-jie Jiang, et al. (2020) | Yes | Yes | Yes | Yes | Yes | Yes | Can’t Tell | Can’t Tell | Yes | Yes | Yes | Yes | Valuable |
| Yuan Fang, et al. (2019) | Yes | Yes | Yes | Yes | Yes | Yes | Can’t Tell | Can’t Tell | Yes | Yes | Yes | Yes | Valuable |
| Manabu Mikamori, et al. (2017) | Yes | Yes | Yes | Yes | Yes | Yes | Yes | Yes | Yes | Yes | Yes | Yes | Valuable |
| Jiahao Gong, et al. (2024) | Yes | Yes | Yes | Yes | Yes | Yes | Can’t Tell | Can’t Tell | No | Yes | Yes | Yes | Valuable |
| Zongqiang Hu, et al. (2023) | Yes | Yes | Yes | Yes | Yes | Yes | Yes | Yes | Yes | Yes | Yes | Yes | Valuable |
| Xiao-Cui Wei, et al. (2021) | Yes | Yes | Yes | Yes | Yes | Yes | Can’t Tell | Can’t Tell | Yes | Yes | Yes | Yes | Valuable |
| Ling Qin, et al. (2020) | Yes | Yes | Yes | Yes | Yes | Yes | Can’t Tell | Can’t Tell | Yes | Yes | Can’t Tell | Yes | Valuable |
| Guohua Lou, et al. (2020) | Yes | Yes | Yes | Yes | Yes | Yes | Can’t Tell | Can’t Tell | Yes | Yes | Yes | Yes | Valuable |
| Guohua Lou, et al. (2015) | Yes | Yes | Yes | Yes | Yes | Yes | Can’t Tell | Can’t Tell | Yes | Yes | Yes | Yes | Valuable |
| Zhao, Q., et al. (2021) | Yes | Yes | Yes | Yes | Yes | Yes | Yes | Yes | Yes | Yes | Yes | Yes | Valuable |
| Lin-Rui Gao, et al. (2024) | Yes | Yes | Yes | Yes | Yes | Yes | Yes | Yes | Yes | Yes | Yes | Yes | Valuable |
| Shuyao Zhang, et al. (2023) | Yes | Yes | Yes | Yes | Yes | Yes | Yes | Can’t Tell | Yes | Yes | Yes | Yes | Valuable |
| Rukun Zang, et al. (2021) | Yes | Yes | Yes | Yes | Yes | Yes | Yes | Yes | Yes | Yes | Yes | Yes | Valuable |
| Yusuo Tong, et al. (2020) | Yes | Yes | Yes | Yes | Yes | Yes | Yes | Yes | Yes | Yes | Yes | Yes | Valuable |
| Qu, B., et al. (2024) | Yes | Yes | Yes | Yes | Yes | Yes | Yes | Yes | Yes | Yes | Yes | Yes | Valuable |
| Makinoya, M., et al. (2024) | Yes | Yes | Yes | Yes | Yes | Yes | Yes | Yes | Yes | Yes | Yes | Yes | Valuable |
| Zhu, T., et al. (2023) | Yes | Yes | Yes | Yes | Yes | Yes | Yes | Yes | Yes | Yes | Yes | Yes | Valuable |
| Liang, Q., et al. (2023) | Yes | Yes | Yes | Yes | Yes | Yes | Yes | Yes | Yes | Yes | Yes | Yes | Valuable |
| Jing, X., et al. (2022) | Yes | Yes | Yes | Yes | Yes | Yes | Yes | Yes | Yes | Yes | Yes | Yes | Valuable |
| Yao, W., et al. (2021) | Yes | Yes | Yes | Yes | Yes | Yes | Yes | Yes | Yes | Yes | Yes | Yes | Valuable |
| Zhang, H., et al. (2020) | Yes | Yes | Yes | Yes | Yes | Yes | Yes | Yes | Yes | Yes | Yes | Yes | Valuable |
| Lin, H., et al. (2020) | Yes | Yes | Yes | Yes | Yes | Yes | Yes | Yes | Yes | Yes | Yes | Yes | Valuable |
| Ziqi Shang, et al. (2023) | Yes | Yes | Yes | Yes | Yes | Yes | Yes | Yes | Yes | Yes | Yes | Yes | Valuable |
| Xianlin Qu, et al. (2023) | Yes | Yes | Yes | Yes | Yes | Yes | Yes | Yes | Yes | Yes | Yes | Yes | Valuable |
| Yang Ganga, et al. (2022) | Yes | Yes | Yes | Yes | Yes | Yes | Yes | Yes | Yes | Yes | Yes | Yes | Valuable |
| [Yuxin He, et al. (2022)](https://pubmed.ncbi.nlm.nih.gov/?term=He+Y&cauthor_id=36206097) | Yes | Yes | Yes | Yes | Yes | Yes | Yes | Yes | Yes | Yes | Yes | Yes | Valuable |
| Jingyu Wang, et al. (2019) | Yes | Yes | Yes | Yes | Yes | Yes | Yes | Yes | Yes | Yes | Yes | Yes | Valuable |
| Shumin Wang, et al. (2020) | Yes | Yes | Yes | Yes | Yes | Yes | Yes | Yes | Yes | Yes | Yes | Yes | Valuable |
